# Acute high-dose irradiation disrupts cell adhesion and Silk-Ovarioid formation in human primary ovarian cells

**DOI:** 10.1186/s13048-025-01932-8

**Published:** 2026-01-02

**Authors:** Spyridon Panagiotis Deligiannis, Tianyi Li, Elisabeth Moussaud-Lamodière, Akos Végvári, Anastasios Damdimopoulos, Darja Lavogina, Kiriaki Papaikonomou, Roman Zubarev, Ganesh Acharya, Agne Velthut-Meikas, Pauliina Damdimopoulou, Andres Salumets, Valentina Di Nisio

**Affiliations:** 1 Division of Obstetrics and Gynaecology, Department of Clinical Science, Intervention and Technology, Karolinska Institutet, Huddinge, Stockholm, SE-14186 Sweden; 2 Department of Gynaecology and Reproductive Medicine, Karolinska University Hospital, Huddinge, Stockholm, SE-14186 Sweden; 3 Celvia CC, Tartu, 50411 Estonia; 4 Department of Obstetrics and Gynaecology, Institute of Clinical Medicine, University of Tartu, Tartu, 51014 Estonia; 5 Department of Obstetrics and Gynaecology, University of Helsinki, Helsinki, 00290 Finland; 6 Department of Oncology and Pathology, Karolinska Institutet, Stockholm, 17177 Sweden; 7 Division of Chemistry I, Department of Medical Biochemistry and Biophysics, Karolinska Institutet, Stockholm, Sweden; 8 Bioinformatics and Expression Analysis core facility, Karolinska Institutet, Huddinge, Stockholm, 14186 Sweden; 9 Department of Haematology and Oncology, Institute of Clinical Medicine, University of Tartu, Tartu, 50406 Estonia; 10 Department of Bioorganic Chemistry, Institute of Chemistry, University of Tartu, Tartu, 50411 Estonia; 11 Department of Women’s and Children’s Health, Karolinska Institutet, Biomedicum, Solnavägen 9, Stockholm, 17177 Sweden; 12 Centre for Foetal Medicine, Karolinska University Hospital, Stockholm, 14183 Sweden; 13 Department of Chemistry and Biotechnology, Tallinn University of Technology, Tallinn, 12618 Estonia

**Keywords:** Human primary ovarian cells, X-ray irradiation, DNA damage, Cell adhesion, p53, Silk-Ovarioids

## Abstract

**Background:**

Radiotherapy is a cornerstone of cancer treatment; however, its effects on healthy ovarian somatic cells remain largely unexplored. This study addresses this gap by investigating how human cortical and medullary primary ovarian cells (cPOCs and mPOCs, respectively) respond to acute, high-dose X-ray exposure in *vitro*.

**Methods:**

Ovarian tissue was obtained from eight patients (aged 23–36 years) undergoing gender-affirming surgery at Karolinska University Hospital in Huddinge, Sweden. The tissue was separated into cortex and medulla and dissociated into cPOCs and mPOCs. Monolayer cultures of cPOCs and mPOCs were exposed to 10 Gy X-rays upon reaching confluency, or left unexposed as paired controls. Following irradiation, cells were assessed for ATP content and mitochondrial dehydrogenase activity, followed by immunofluorescence staining, bulk RNA sequencing (Illumina Stranded mRNA Prep Ligation protocol; sequencing on the Illumina NovaSeq 6000 platform), bulk proteomic analysis (liquid chromatography–tandem mass spectrometry), and a functional assay for assessing their ability to form 3D Silk-Ovarioids.

**Results:**

While irradiation did not significantly affect cell viability, immunofluorescence analyses revealed alterations in DNA damage response, apoptosis, and cell cycle regulation. Transcriptomic analysis showed minimal changes at 1 h post-irradiation in both cPOCs and mPOCs. However, marked shifts in transcriptomic profiles were observed at 4 h (2,810 and 2,540 DEGs in cPOCs and mPOCs, respectively) and at 24 h (2,462 and 2,802 DEGs, respectively), including upregulation of the p53 pathway and downregulation of MYC targets, E2F targets, the G2/M checkpoint, and the mTORC1 pathway. At the proteomic level, differentially expressed proteins associated with cell adhesion, focal adhesion, and cadherin binding were detected at 24 h post-irradiation. Functionally, irradiated cells demonstrated an impaired capacity to self-organize into 3D Silk-Ovarioids, indicating compromised cell–cell adhesion.

**Conclusion:**

These findings reveal a novel mechanism by which radiotherapy may damage ovarian tissue independently of follicular loss, underscoring the need for targeted strategies to preserve somatic cell function in fertility preservation protocols.

**Supplementary Information:**

The online version contains supplementary material available at 10.1186/s13048-025-01932-8.

## Introduction

The rising global incidence of cancer affects nearly one million women of reproductive age every year [1]. Ionizing irradiation or radiotherapy (RT), often used in combination with chemotherapy and surgery, is a cornerstone of cancer therapy and is applied to approximately 30–50% of patients [2]. The widespread use of RT is attributed to its low overall cost - accounting for only 5% of total cancer care expenditures - and its significant contribution to cancer survival, accounting for approximately 40% of all cancer cures [3, 4].

While RT effectively targets cancer cells, it also impacts normal non-malignant cells, increasing the risk of long-term collateral damage and compromising tissue integrity. Radiation-induced tissue damage involves multiple processes, such as inflammation, oxidative stress, cell death, extracellular matrix (ECM) remodeling and fibrosis [5]. Irradiation exposure directly or indirectly (i.e., *via* reactive oxygen species production) damages cellular DNA, thereby activating DNA repair pathways. The cellular responses triggered by DNA damage are primarily driven by the tumour suppressor protein p53. This transcription factor can halt the cell cycle to allow repair or induce apoptosis, both mechanisms serving to preserve genomic integrity at the tissue and organ levels [6, 7]. Additionally, c-Myc plays a dual role in response to irradiation-induced damage. It is essential for the activation of ATM-dependent DNA damage responses, facilitating the recruitment of H2AX (the histone guardian of the genome) and triggering checkpoint mechanisms that either arrest cell proliferation or induce p53-dependent apoptosis [8]. However, c-Myc can also disrupt the repair of double-strand breaks and the G1/S checkpoint, while promoting S-phase entry, leading to genomic instability [9]. These adverse effects have been evidenced post-RT in various organs [10–12].

The ovary is one of the organs particularly sensitive to RT-induced side effects. Altogether, these damages may contribute to long-term side effects in women of reproductive age [13]. Ovaries consist of two distinct compartments: the outer cortex and the inner medulla. The cortex, about 1 mm thick, contains small pre-antral follicles, including the dormant ones that constitute the ovarian reserve. The medulla has a smoother texture and houses larger secondary and antral follicles. Single-cell RNA-sequencing has revealed distinct cell population profiles in the medulla compared to the cortex, with key differences observed in the theca cells and high diversity among the granulosa cells (GC) [14, 15]. This is likely due to the presence of multiple GC sub-populations from antral follicles in the medulla [16]. Together, these layers support important ovarian functions such as folliculogenesis and steroidogenesis.

Despite the cellular complexity of ovarian tissue, previous studies have mostly focused on irradiation-related effects on follicles. In the early stages of folliculogenesis, GC exhibit high proliferative activity, while the oocyte is highly sensitive to stressors and toxicants. This vulnerability makes early-stage follicles particularly susceptible to radiation damage, potentially leading to subfertility and, in extreme cases, to premature ovarian insufficiency (POI) [17–20]. The extent of damage depends on different factors, including patient’s age, radiation dose, and anatomical site of RT [21]. For instance, a single dose of 2 Gy (Gy) irradiation in the pelvic area can damage over 50% of the ovarian reserve, while doses of 10 Gy may cause cessation of ovarian function in adults [22]. Clinically, doses of 10–20 Gy and 4–6 Gy are associated with POI in children and adults, respectively [23].

In 2021, based on stringent systematic literature review, the PanCareLIFE Consortium published guidelines recommending fertility preservation strategies, including oocyte, embryo and ovarian tissue cryopreservation, for female oncology patients of paediatric and reproductive age. The guidelines strongly recommend counselling patients regarding fertility preservation options in cases of RT with ovary in the field [24]. Albeit most research focuses on follicle-related damage, study investigating the impact of RT on the ovaries should extend beyond follicle survival. In fact, RT treatments can also significantly affect the ovarian stroma, which has been observed to shrink following irradiation due to fibrosis and atrophy [25]. Damage to ovarian stromal cells in both the cortex and the medulla can further contribute to follicular loss, as these cells are unable to produce antioxidants and anti-apoptotic factors such as Bcl-2 [18], and may also alter follicular ECM microenvironment [18, 26]. Moreover, RT can cause small vessel damage, leading to thrombosis and the deposition of fibrin in vessels, thereby further compromising ovarian function [25]. The involvement of different ovarian cell types in post-RT organ damage urges the use of three-dimensional (3D) tissue and organ models in future studies of X-ray induced ovarian damage.

In recent years, 3D ovary models using primary cells have revolutionized studies on ovarian physiopathology. Among the numerous research on both animal and human models, our recent study developed and characterized a long-term cultured 3D ovarian model, named Silk-Ovarioids [27]. Silk-Ovarioids can be generated from both cortical and medullary ovarian cells and contain key ovarian somatic cell types. Additionally, they exhibit low intra-batch variability during long-term culture, making them a reliable model for investigating RT-dependent ovarian impact.

In the current study, we investigated the effects of ionizing radiation on human primary ovarian cells (POCs) in vitro. POCs were isolated from the ovarian cortex (cPOCs) or medulla (mPOCs) and exposed to a single clinically relevant 10 Gy dose of X-ray. Following irradiation, we assessed cellular ATP levels and mitochondrial reductase activity over a 7-day period, interrogated the activation of canonical pathways related to irradiation-induced damage and repair, analysed changes in cellular transcriptomic and proteomic profiles, and evaluated the ability of ovarian cells to form Silk-Ovarioids.

## Materials and methods

### Study participants

**Fig. 1:**
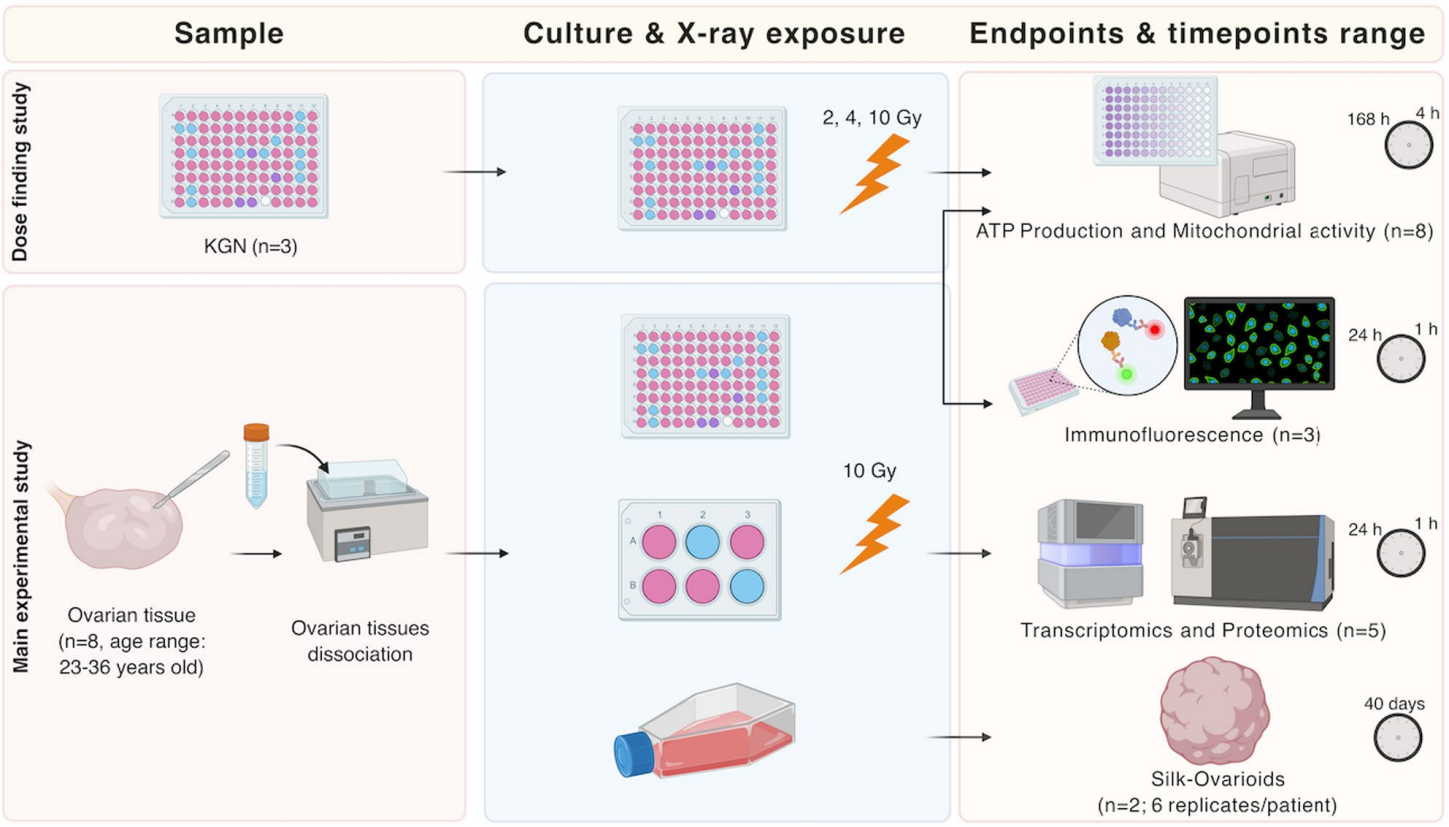
Schematic overview of the experimental workflow. Experimental setup of in vitro culture of ovarian primary cells, X-ray exposure and endpoints of the study. ATP, adenosine triphosphate; cPOCs, cortex-derived primary ovarian cells; Gy, Gray; mPOCs, medulla-derived primary ovarian cells. Created with BioRender.com

The overall experimental flow is demonstrated in Fig. 1. Human ovarian tissue was obtained from patients undergoing gender-affirming surgery (*n* = 8; age range: 23–36 years) at Karolinska University Hospital in Huddinge, Sweden. Informed consent was obtained in accordance with the Declaration of Helsinki. During surgery, bilateral oophorectomy was performed, and the ovarian tissue was immediately transferred to the laboratory within 10 min in Dulbecco’s phosphate buffered saline (DPBS) supplemented with calcium, magnesium, glucose, and sodium pyruvate (Life Technology, UK). The ovarian medulla was carefully trimmed and separated from the cortex (depth 1–1.5 mm). Both were subsequently cryopreserved using a slow-freezing protocol [28]. Briefly cortical and medullary tissues were cut into pieces of 5 × 5 × 1 mm in size and equilibrated on ice for a maximum of 30 min in a slow-freezing medium containing 7.5% ethylene glycol (Sigma-Aldrich, USA), 33.9 mg/ml sucrose (Sigma-Aldrich, USA) and 10 mg/ml human serum albumin (HSA, Vitrolife, Sweden) diluted in DBPS (Thermo Scientific, USA) on a shaker. Afterwards, the pieces were transferred into Nunc™ CryoTube™ Vials (ThermoFisher Scientific, USA) containing 1 mL of slow-freezing medium and placed into a controlled-rate freezer (Kryo 230 − 1.7, Planer PLC). Tissue samples were later thawed and used in further experiments.

### KGN cell culture

KGN cells, a human granulosa cell line derived from an ovarian tumour [29], were selected for irradiation dose optimization due to their similarity in proliferation rates and transcriptomics profile to POCs [30]. The KGN cell line was obtained from the RIKEN Cell Bank (RBRC-RCB1154, Japan). Detailed information on KGN cell culture settings is described in Supplementary File 1.

### Ovarian tissue dissociation and primary ovarian cell culture

For tissue dissociation, cryopreserved/thawed ovarian cortex and medulla were separately digested into a single cell suspension using a mechanical and enzymatic method, as previously described [15]. Detailed information on ovarian tissue dissociation and primary ovarian cell culture settings is described in Supplementary File 1.

### KGN and POCs X-ray irradiation

KGN, cPOCs and mPOCs were irradiated in a CIX2 X-ray irradiator cabinet (Xstrahl, Surrey, England) with a single dose of 2, 4 or 10 Gy, delivered at a dose rate of 1.33 Gy/min (dosimetry uncertainty ca. 1.7%) at 195 kV and 10 mA at the X-ray Irradiation Core Facility, Karolinska Institutet (Huddinge, Sweden). Focus skin distance (FSD) to the flask/plate was 40 cm. External filtration giving an HVL of 9.0436 mm Al was applied by adding a 3.0 mm Al filter. Cells were also rotated during irradiation to ensure a more uniform dose distribution using a rotating table. Sham controls were maintained under identical conditions, except for exposure to radiation, for the same duration. To determine the optimal dose for POCs, KGN cells were exposed to a single dose of 2, 4, or 10 Gy. The maximum dose of 10 Gy was based on the PanCareLIFE Consortium guidelines [24]. Cellular ATP levels, activity of mitochondrial dehydrogenases and DNA damage on KGN cells were assessed post-irradiation, leading to the selection of 10 Gy for subsequent cPOCs and mPOCs experiments performed immediately upon reaching confluency.

### Measurement of intracellular ATP level and mitochondrial dehydrogenases activity

Intracellular ATP levels in KGN (*n* = 3), cPOCs (*n* = 8) and mPOCs (*n* = 8) were assessed within 7 days post-irradiation using the CellTiter-Glo^®^ assay (Promega, USA), following the manufacturer’s instructions. Irradiation-induced metabolic changes in mitochondria were quantified using the Cell Proliferation Kit I (MTT) (Roche Diagnostics, Germany) following manufacturer’s instructions. Detailed information on CellTiter-Glo^®^ assay and Cell Proliferation Kit I (MTT) is described in Supplementary File 1.

### Immunofluorescence staining

Irradiated KGN (*n* = 1), cPOCs (*n* = 3) and mPOCs (*n* = 3) were fixed with 4% paraformaldehyde (PFA; Sigma-Aldrich, USA) at 1 h, 4 h and 24 h post-irradiation for immunofluorescence staining. Non-irradiated controls were cultured and fixed alongside the 1 h irradiated samples. Cells were permeabilized with blocking buffer, followed by overnight incubation in primary antibody. After incubation, cells were washed and incubated in secondary antibody for 1 h, followed by nuclear counterstain incubation with 4’,6-diamidino-2-phenylindole dihydrochloride (DAPI; Thermo Fischer Scientific, USA). Slides were mounted using DAKO Fluorescence Mounting Medium (Agilent Technologies, USA). Detailed information on the immunofluorescence staining, antibody dilutions, microscope imaging and quantification is described in Supplementary File 1.

### RNA extraction and library preparation

RNA extraction was performed from cPOCs (*n* = 5) and mPOCs (*n* = 5) using the RNeasy Micro Kit (Qiagen, Germany), following the manufacturer’s instructions. The extracted RNA was used for library preparation using the Illumina Stranded mRNA Prep Ligation protocol (Illumina, USA). Detailed information on RNA extraction and library preparation is described in Supplementary File 1.

### RNA sequencing and data analysis

RNA sequencing (paired-ends, 2× 150 bp) was performed on the Illumina NovaSeq6000 platform at the National Genomics Infrastructure (NGI), SciLife Lab, Sweden. We aimed at 50 M reads per sample, and approximately an average of 42 M read per sample was obtained after sequencing. *Fastq* files were trimmed using Trim Galore (version 0.6.1) to remove the low-quality reads, then mapped to the human genome (hg38) using the STAR aligner (version 2.7.11a). Bam files were aligned using *featureCount* function in subread package (version 2.0.3) and annotated using UCSC gtf file (available at https://emea.support.illumina.com/sequencing/sequencing_software/igenome.html). The trimming, mapping, and alignment pipeline was provided in a snakemake file (snakemake version 5.10.0). Differential expression analysis was performed in R (version 4.3.2) [31] via RStudio using DESeq2 package. Comparisons were made for 24 h control vs. 1 h control; 1 h and 4 h irradiated vs. 1 h control; and 24 h irradiated vs. 24 h control separately for cPOCs and mPOCs dataset. After quality control, two outliers in cPOCs (i.e., one sample from 24 h control and one from 24 h irradiated group) were removed from the downstream analysis. Differentially expressed genes (DEGs) with false discovery rate (FDR) < 0.05 were considered statistically significant. Gene pattern analysis of DEGs was performed in all the comparisons in cPOCs and mPOCs separately using DEGreport package [32]. Subsequently, the signalling pathways related to the identified gene clusters were inferred using Gene Ontology (GO) over-representation analysis through clusterProfiler [33, 34]. Affected biological pathways were identified using Gene set enrichment analysis (GSEA) against the MSigDB [35] hallmark gene set on all expressed genes ranked by log_2_ fold change through clusterProfiler [33, 34], pathview [36], DOSE [37] and apeglm [38] packages. ComplexHeatmap [39, 40] package was used for heatmap plotting. The pathway activity scores in each sample were predicted using gene set variation analysis (GSVA) on cpm-normalised and log-transformed data against Hallmark gene sets [41]. Analysis codes are available at https://github.com/tialiv/X-Ovary .

### Proteomics sample preparation and data analysis

Irradiated cPOCs (*n* = 5) and mPOCs (*n* = 5), along with their respective controls, were snap-frozen and thawed on ice and lysed with 10 µL of 8 M urea, followed by sonication (5 min) in water bath. Detailed information on the proteomics sample preparation and Liquid chromatography-tandem mass spectrometry data acquisition is described on Supplementary File 1.

Raw data files were processed using Proteome Discoverer v3.0 (Thermo Fisher Scientific) with the MS Amanda v2.0 search engine, referencing the human protein database (SwissProt, 20,022 entries, downloaded on February 9, 2023). Up to two missed cleavage sites were allowed for full tryptic digestion, with precursor and fragment ion mass tolerances set to 10 ppm and 0.02 Da, respectively. Carbamidomethylation of cysteine was considered a fixed modification, while oxidation of methionine, deamidation of asparagine and glutamine, and TMT6plex (+ 229.163 Da) on lysine and peptide N-termini were treated as dynamic modifications. The initial search results were filtered to a 5% FDR using the Percolator node in Proteome Discoverer, with quantification based on the intensities of the reporter ions.

Proteomics data was analysed using R (version 4.3.2) and RStudio. One sample in cPOCs 1 h irradiated group and one sample mPOCs 4 h irradiated group were missing due to the low amount of protein extracted. After removing missing values, data was median-normalised, batch effects removed using *removeBatchEffect* function in limma package [42], and log_2_-transformed before differential expression analysis. Fold changes were calculated by subtracting the mean of the irradiated groups from the mean of the control groups. Comparisons of 1 h and 4 h irradiated vs. 1 h control, and 24 h irradiated vs. 24 h control were performed separately in cPOCs and mPOCs samples, followed t-test and adjustment for multiple comparisons using Benjamini-Hochberg method. Differentially expressed proteins (DEPs) with FDR < 0.1 were considered statistically significant. The affected pathways were inferred using g: Profile web server against Gene Ontology database [43].

### Formation and culture of Silk-Ovarioids

Monolayer cultures of cPOCs (*n* = 2) and mPOCs (*n* = 2) were detached with TrypLE (ThermoFisher Scientific, USA) 24 h post-irradiation and utilised for Silk-Ovarioids formation, as previously described [27]. Detailed information on the experimental approach used for the formation of Silk-Ovarioids is described in the Supplementary File 1.

### Statistics

Intracellular ATP levels and mitochondrial dehydrogenases activity data were processed using GraphPad prism v10. Parametric data significance was assessed by one-way ANOVA. Protein expression of DNA-damage, apoptosis and cell cycle markers data was analysed using the Shapiro-Wilk normality test performed using the dplyr R package to assess data distribution. For non-parametric data, statistical significance was determined using the Kruskal-Wallis test, followed by the Benjamini-Hochberg post-hoc test from the dunn.test R package.

## Results

### Overall experimental flow

The study aimed to investigate the effects of X-ray irradiation on primary ovarian cells. After a dose-finding study in KGN cells, a total dose of 10 Gy was applied to cPOCs and mPOCs, and intracellular ATP levels and activity of mitochondrial dehydrogenases were assessed over a 7-day culture period post-irradiation. Transcriptomic and proteomic analyses were conducted at 1 h, 4 h and 24 h post-irradiation with non-irradiated controls sampled at 1 h and 24 h. Immunofluorescence staining was performed in cPOCs and mPOCs at 1 h, 4 h and 24 h post-irradiation, and in non-irradiated 1 h controls. Finally, the functionality of irradiated cPOCs and mPOCs was tested by evaluating their ability to form Silk-Ovarioids (Fig. 1).

### Dose-finding screening on KGN model

To identify a clinically relevant and sublethal dose of irradiation, KGN cells (*n* = 3) were exposed to 2, 4, and 10 Gy of X-ray irradiation. Subsequently, intracellular ATP levels and activity of mitochondrial dehydrogenases were measured at 4 h, 24 h, 48 h, 72 h, 120 h, and 168 h post-irradiation. No significant differences across all doses and timepoints were detected (Supplemental Fig. S1). Additionally, we qualitatively assessed DNA damage after 2, 4, and 10 Gy X-ray exposure by immunolabelling γ-H2AX, a marker of DNA double-strand breaks. γ-H2AX staining was absent or sparse at all timepoints following 2 Gy and 4 Gy exposure, similar to the non-irradiated control (Supplemental Fig. S2). In contrast, 10 Gy irradiation induced a clear increase in γ-H2AX-positive cells at 1 h, which progressively declined over time and returned to control levels by 24 h (Supplemental Fig. S2).

### Intracellular ATP levels and mitochondrial dehydrogenases activity of POCs are not affected by irradiation

Based on these results, a 10 Gy dose was selected for experiments involving cPOCs and mPOCs (*n* = 8). Intracellular ATP levels and activity of mitochondrial dehydrogenases were monitored at 4 h, 24 h, 72 h, 120 h, and 168 h post-exposure, with unexposed cells at 4 h and 120 h serving as controls. In both cPOCs and mPOCs, ATP levels remained stable throughout the culture period (Supplemental Fig. S3A) and activity of mitochondrial dehydrogenases showed no significant changes across the 168 h post-irradiation culture period (Supplemental Fig. S3B).

### Immunofluorescence analysis of canonical pathways induced by irradiation

Since no significant changes were observed in ATP production or mitochondrial dehydrogenase activity, we performed immunofluorescence staining to assess the activation of canonical pathways associated with irradiation-induced damage [44]. Specifically, we examined markers related to DNA damage, apoptosis and cell cycle regulation in both cPOCs and mPOCs at 1 h, 4 h and 24 h post-irradiation.

#### Effects of irradiation on DNA damage in cPOCs and mPOCs

To evaluate the DNA damage response, we analysed the expression of phosphorylated Chk1 (Ser345) (p-Chk1) and γ-H2AX (Ser139) in cPOCs and mPOCs (Supplemental Fig. S4). In cPOCs, both p-Chk1 and γ-H2AX levels were significantly upregulated at 1 h post-irradiation (*p* < 0.001) and began to decline by 4 h, while remaining significantly elevated (*p* < 0.001) relative to the non-irradiated control. Both markers returned to the baseline levels by 24 h post-irradiation (Supplemental Fig. S4A). In contrast, mPOCs displayed a delayed response, with p-Chk1 and γ-H2AX expression remaining at baseline levels 1 h post-irradiation. Their levels peaked significantly at 4 h post-irradiation (*p* < 0.001) and returned to baseline by 24 h (Supplemental Fig. S4B). This suggests a later transient activation of DNA damage response in mPOCs compared to cPOCs.

#### Effects of irradiation on the activation of apoptosis in cPOCs and mPOCs

To assess apoptotic activation, we evaluated levels of cleaved caspase-3 (cl-Cas3), phosphorylated p53 (Ser15) (p-p53), and Bcl-2 (Supplemental Fig. S5). In cPOCs, p-p53 showed a non-significant increase at 1 h, followed by a significant decrease at 4 h (*p* < 0.05) and 24 h (*p* < 0.001) post-irradiation relative to the non-irradiated control. cl-Cas3 remained at baseline at 1 h and 4 h and significantly decreased at 24 h (*p* < 0.01). Conversely, Bcl-2 expression significantly increased at 1 h (*p* < 0.05) and 4 h (*p* < 0.001), returning to baseline by 24 h post-irradiation (Supplemental Fig. S5A).

In mPOCs, p-p53 showed no significant changes across timepoints. cl-Cas 3 was increased significantly at 1 h (*p* < 0.01), followed by a gradual decrease until returning to baseline levels at 24 h post-irradiation. Similarly, Bcl-2 was significantly elevated at 1 h (*p* < 0.001) and 4 h (*p* < 0.001) but returned to baseline levels by 24 h post irradiation (Supplemental Fig. S5B). These results suggest that cPOCs exhibit an early protective response to irradiation through prolonged Bcl-2 activation, increasing from 1 h to 4 h timepoints. In contrast, mPOCs initiated apoptosis with elevated cl-Cas 3 levels, accompanied by simultaneous activation of anti-apoptotic Bcl-2, resulting in a transient apoptotic response.

#### Effects of irradiation on cell cycle regulation in cPOCs and mPOCs

We further assessed key cell cycle regulators, including cyclin D1, cyclin E and phosphorylated p21 (Ser146) (p-p21) in both cPOCs and mPOCs (Supplemental Fig. S6). In cPOCs, cyclin D1 remained unchanged throughout the culture period, whereas cyclin E remained significantly elevated (1 h, *p* < 0.01; 4 h, *p* < 0.05; 24 h, *p* < 0.05) relative to the non-irradiated control. Meanwhile, p-p21 levels were significantly increased at 1 h (*p* < 0.05), before returning to baseline levels at 4 h and 24 h (Supplemental Fig. S6A). In mPOCs, both cyclin D1 and cyclin E showed no significant changes at 1 h and 4 h but were significantly decreased at 24 h (*p* < 0.01) relative to the non-irradiated control. Notably, p-p21 levels remained significantly elevated throughout the 24 h (1 h, *p* < 0.001; 4 h, *p* < 0.01; 24 h, *p* < 0.001) post-irradiation period (Supplemental Fig. S6B). These results suggest that cPOCs efficiently recover from irradiation-induced stress, while mPOCs undergo prolonged cell cycle arrest due the persistent elevation of p-p21, likely allowing DNA repair.

### Transcriptomic analysis identified distinct irradiation related gene expression profiles in cPOCs and mPOCs

To investigate the impact of X-ray irradiation on the transcriptome, bulk RNA-sequencing was performed on cPOCs (*n* = 5) and mPOCs (*n* = 5) at 1 h, 4 h and 24 h post-irradiation, along with the non-irradiated controls at 1 h and 24 h. We first assessed potential transcriptomic differences between the two baseline controls. Principal components analysis (PCA) revealed distinct transcriptomic profile between the 1 h and 24 h controls in both cPOCs (Supplemental Fig. S7A) and mPOCs (Supplemental Fig. S7B). Subsequently, we performed differential expression analysis (DEA) and predicted the irradiation-affected signalling using GSEA analysis. Our results revealed upregulation of cell-cycle-related transcripts, including E2F target and G2M checkpoint genes, in the 24 h control samples compared to 1 h controls (Supplemental Fig. S7). These findings indicate that the observed effects were mainly related to culture progression. On these bases, we decided to compare the irradiated samples at early timepoints (1 h and 4 h) to the non-irradiated control at 1 h, while the longer post-irradiation timepoint (24 h) was compared to the respective 24 h controls.

**Fig. 2:**
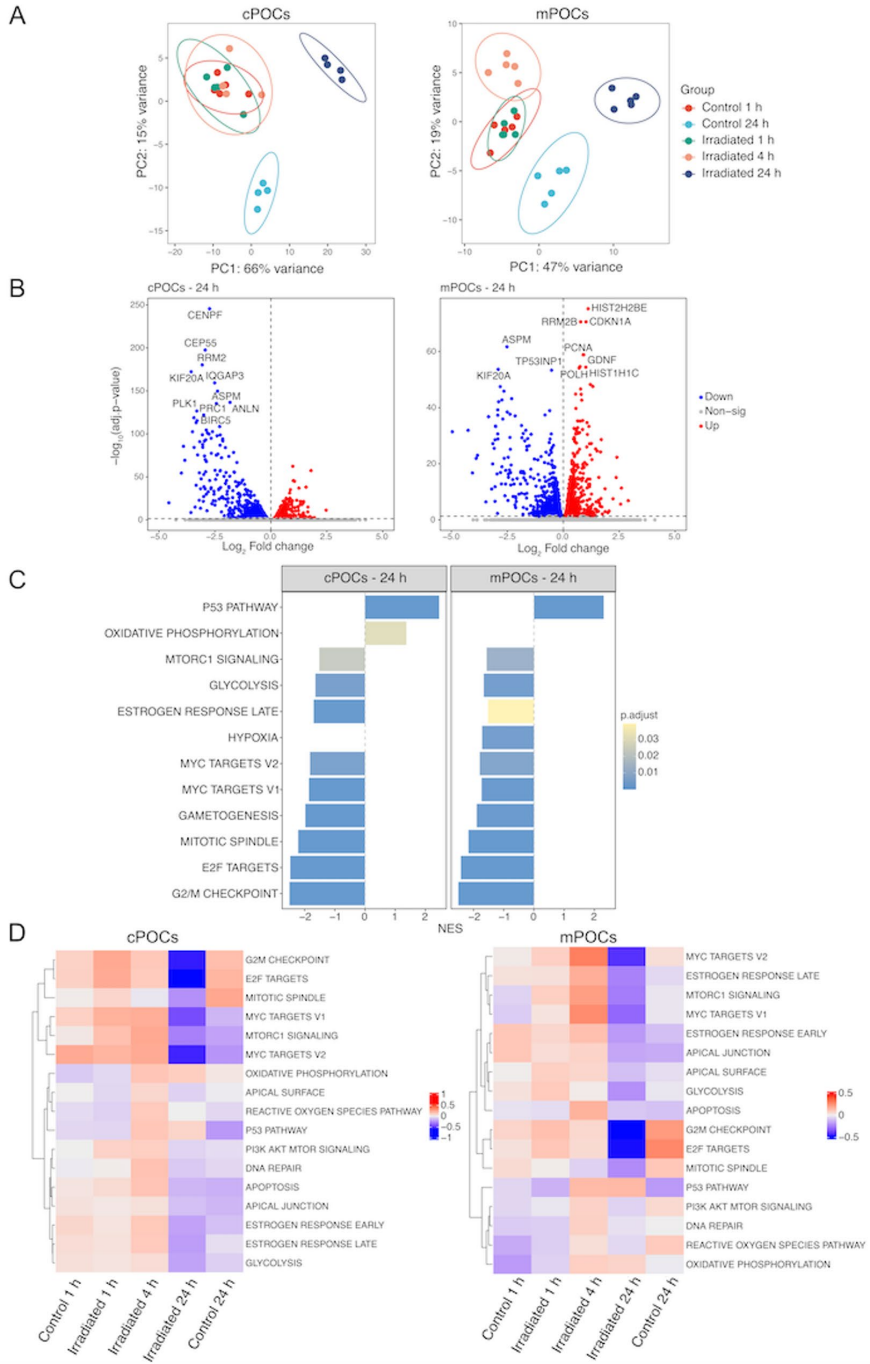
Irradiation induced changes in the transcriptomic profiles of cPOCs and mPOCs. **A **PCA of the top 500 highly variable genes after removing batch effects in the cPOCs and mPOCs. Two outliers from cPOCs groups were removed from the dataset (in 24 h control and irradiated groups). **B** Volcano plot showing the up- and downregulated DEGs at 24 h post-irradiation in cPOCs and mPOCs. The top 10 significant DEGs were annotated in the plots. Colour scheme: red indicates gene upregulation, blue indicates gene downregulation, and grey indicates no significant differences in gene expression. **C** Significantly enriched hallmark gene sets predicted by GSEA using all expressed genes ranked by log_2_ fold changes at 24 h post-irradiation. NES is shown on the x axis. **D** Heatmap of pathway enrichment scores of selected hallmark gene sets predicted by GSVA at 1 h, 4 h and 24 h in cPOCs and mPOCs. cPOCs, cortex-derived primary ovarian cells; GSEA, gene set enrichment analysis, GSVA, Gene set variation analysis; mPOCs, medulla-derived primary ovarian cells; NES, Normalised enrichment score; PCA, Principal component analysis

We investigated the effects of irradiation after stratifying the dataset by timepoints. After quality control, two outliers in cPOCs (in 24 h control and irradiated groups) were removed from downstream analysis. PCA of all samples revealed a clear separation by timepoints and X-ray exposure (Fig. 2A). Due to the predominant transcriptomic difference in the 24 h samples, the effects at early timepoints could not be clearly visualized in the overall PCA. For this reason, we plotted the early timepoints (1 h and 4 h) and late timepoint separately (Supplemental Fig. S8A, B). The stratified PCA demonstrated an early transcriptomic shift already at 4 h post-irradiation in both cPOCs and mPOCs (Supplemental Fig. S8A, B). Consistent with these results, DEA revealed minor differences between the irradiated samples and their corresponding controls at 1 h post-irradiation (3 DEGs in cPOCs and 1 in mPOCs; Supplemental Table S1). More pronounced changes emerged at 4 h with 2,810 and 2,540 DEGs identified in cPOCs and mPOCs, respectively. These transcriptomic alterations persisted at 24 h with 2,462 DEGs in cPOCs and 2,802 in mPOCs (Supplemental Table S1). Among these, genes related to cell cycle and p53 signalling, such as *CENPF*, *PLK1*, *CDKN1A*, *PCNA*, and *TP53INP1*, were highly significant at 24 h post-irradiation (Fig. 2B).

To assess whether cPOCs and mPOCs responded differently to irradiation, we compared timepoint-specific DEGs at 1 h, 4 h and 24 h post-irradiation (Supplemental Fig. S9). After 1 h, the effects were unique to each group, while substantial numbers of common DEGs were observed at 4 h and 24 h post-irradiation, where 1,384 and 1,451 DEGs shared between cPOCs and mPOCs, respectively (Supplemental Fig. S9). We further inferred the biological processes related to these common genes through GO over-representation analysis. At 4 h post-irradiation, GOs related to transcription process (e.g., nuclear speckles involved in splicing and DNA-binding transcription repression) and histone modifications were enriched (Supplemental Fig. S9A). At 24 h, the common pathways affected in cPOCs and mPOCs were predominantly cell cycle-related, including mitotic cell cycle phase transition, cell cycle checkpoint signalling and sister chromatid segregation (Supplemental Fig. S9B). Alongside these shared effects, cPOCs and mPOCs also displayed unique responses at different timepoints. In cPOCs, these effects were mainly related to autophagy and cell cycle, i.e., ubiquitin ligase complex and autophagosome organisation at 4 h, and DNA replication and mitotic spindle function at 24 h (Supplemental Fig. S9). Interestingly, cell-cell communication pathways were affected at the transcriptomic level only in mPOCs, as shown by the enriched GO terms related to collagen homeostasis and extracellular matrix organisation at 4 h, and cell-cell junction, focal and cell-substrate adhesion at 24 h post-irradiation (Supplemental Fig. S9A).

To capture the subtle changes induced by X-ray irradiation, especially at 1 h post-irradiation, we performed GSEA against the hallmark gene sets using all expressed genes ranked by log_2_ fold change at each timepoint. Furthermore, to better assess the time-specific changes, we performed GSVA, obtaining a pathway activity score for each timepoint. Our results showed that MYC targets, E2F targets, and G2M checkpoint transcripts were upregulated in both cPOCs and mPOCs, although significance was reached in cPOCs at 1 h post-irradiation (Fig. 2D, Supplemental Fig. S8C). As expected, upregulation of p53 pathways was displayed at 4 h and 24 h post-irradiation in both cPOCs and mPOCs (Fig. 2C, D, Supplemental Fig. S8C), suggesting the activation of DNA damage signalling on a transcriptomic level. Samples at 4 h post-irradiation showed a higher pathway activity score in the PI3K-AKT-mTOR signalling, DNA repair, and estrogen response in cPOCs and mPOCs (Fig. 2D). Following the progression of time, the transcriptomic changes became more prominent at 24 h post-irradiation, where downregulation of cell cycle related gene sets and glycolysis were observed (Fig. 2C, D).

### Gene pattern analysis and GO enrichment revealed effects on DNA damage, and chromosome segregation in cPOCs post-irradiation

**Fig. 3:**
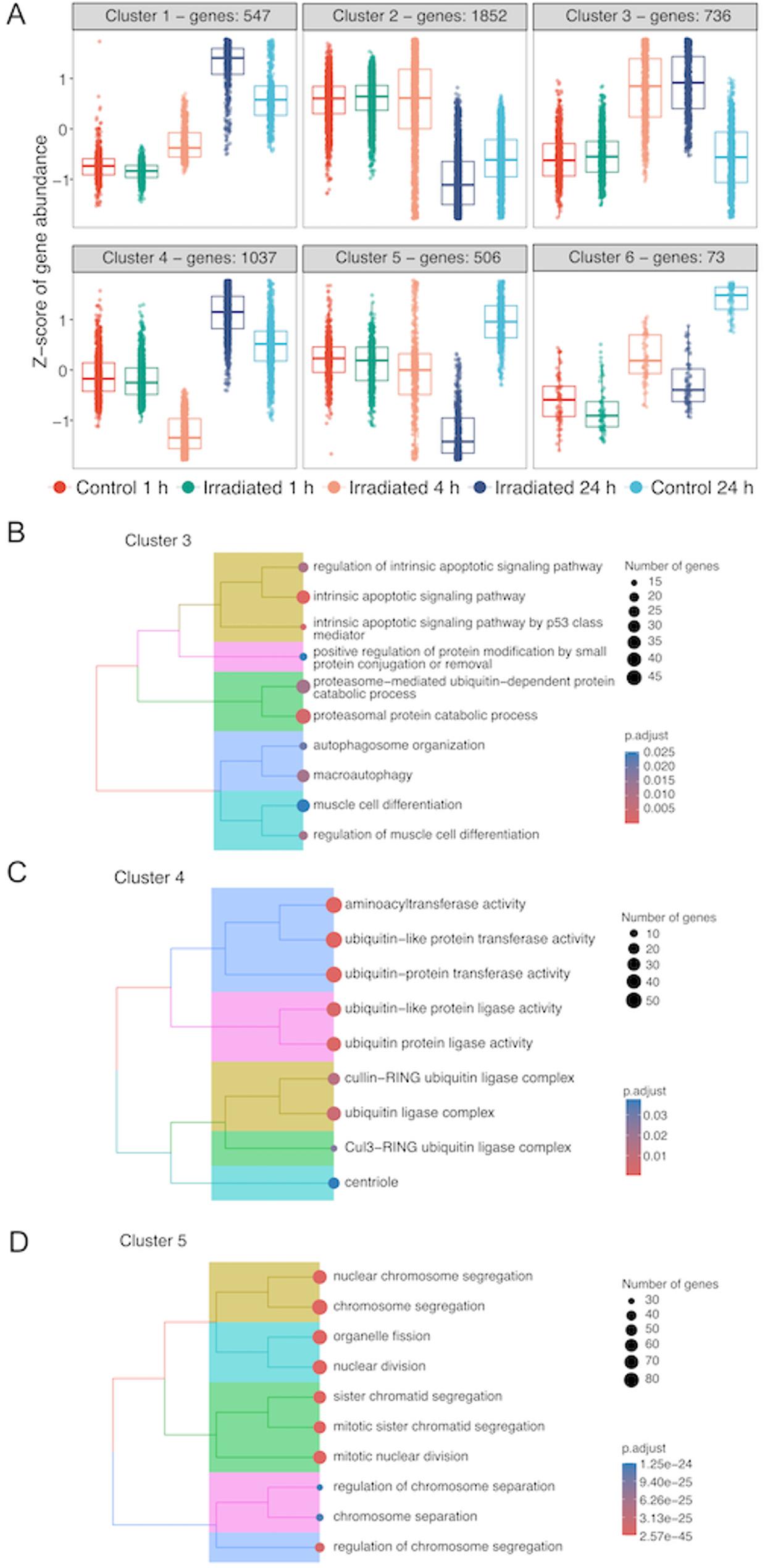
Gene pattern analysis in cPOCs.** A** Gene pattern analysis using pooled DEGs from all group-wise comparisons. Z-scores of gene abundance is displayed on y-axis. Tree plot of top 10 significantly enriched GO terms using DEGs identified in (**B**) cluster 3, (**C**) cluster 4 and (**D**) cluster 5. GO terms were clustered using ward. D method based on the similarity of genes in the pathways and the position of the pathway in the GO network. The size of the dots represents the number of genes enriched in the GOs. Colour of the dots indicates the adjusted p-value. cPOCs, cortex-derived primary ovarian cells; DEGs, differentially expressed genes; GO, gene ontology

**Fig. 4:**
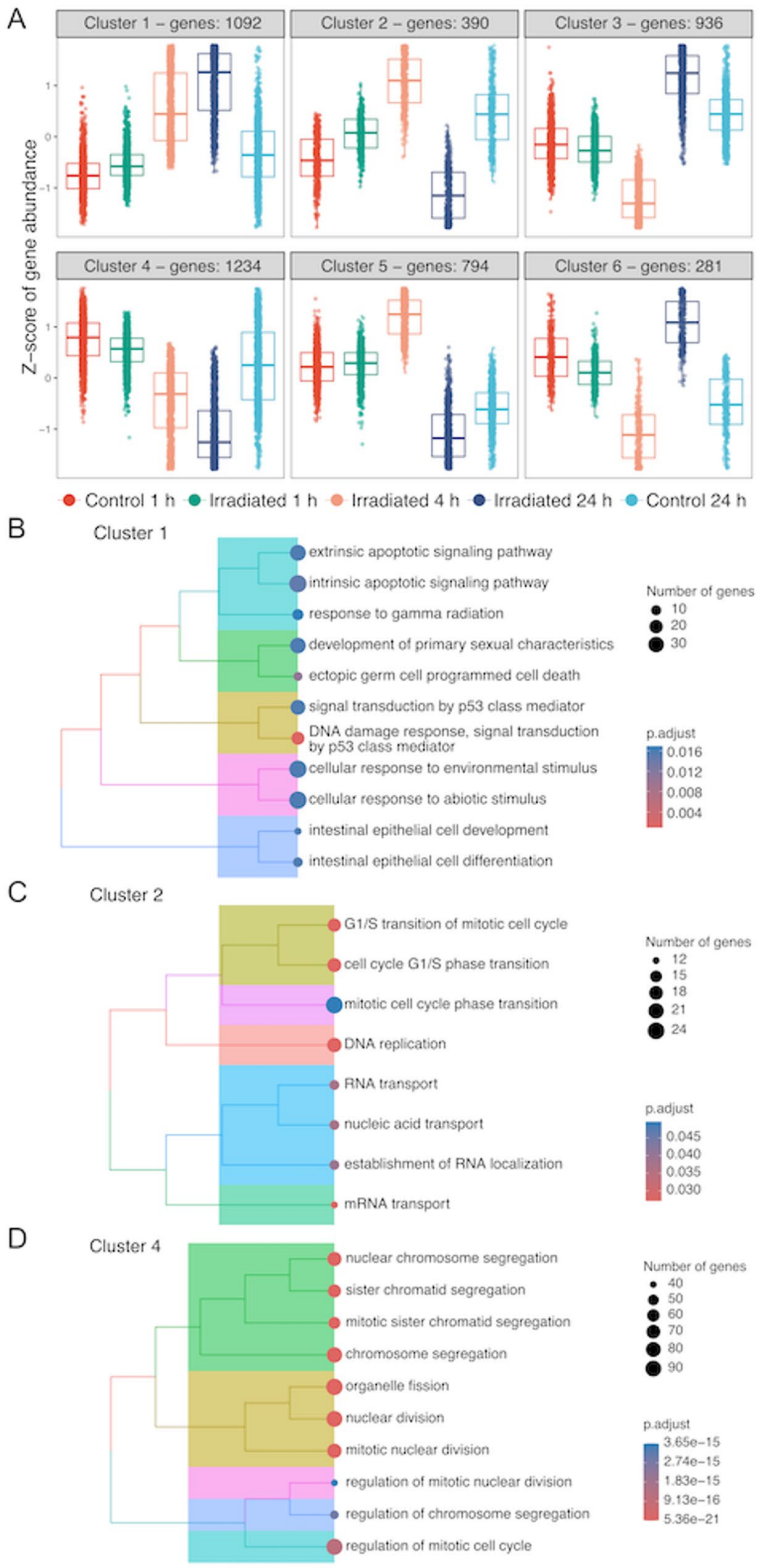
Gene pattern analysis in mPOCs.** A** Gene pattern analysis using pooled DEGs from all group-wise comparisons. Z-scores of gene abundance is displayed on y-axis. Tree plot of top 10 significantly enriched GO terms using DEGs identified in (**B**) cluster 1, (**C**) cluster 2, and (**D**) cluster 4. GO terms were clustered using ward. D method based on the similarity of genes in the pathways and the position of the pathway in the GO network. The size of the dots represents the number of genes enriched in the GOs. Colour of the dots indicates the adjusted *p*-value. DEGs, differentially expressed genes; GO, gene ontology; mPOCs, medulla-derived primary ovarian cells

To assess whether X-ray induced transcriptomic changes could follow a consistent pattern over the 24 h of recovery, we considered DEGs identified from all comparisons as potential targets to investigate whether gene patterns exist across timepoints after irradiation. To focus our attention on the most representative irradiation-related gene patterns, we set a maximum of 6 distinct gene clusters in both cPOCs and mPOCs and performed the gene pattern analysis (Figs. 3 and 4). Among these gene clusters, we selected clusters 3, 4, and 5 where the two baseline controls (1 h and 24 h) exhibited similar expressions, while the irradiated groups showed substantial deviation (Fig. 3A). In cluster 3, genes related to apoptosis signalling and autophagy were upregulated at 4 h and 24 h post-irradiation (Fig. 3A, B, Supplemental Table S2). Genes in cluster 4 exhibited a downregulation at 4 h, followed by an upregulation at 24 h post-irradiation (Fig. 3A). GO over-representation analysis showed that this unique pattern was associated with ubiquitin activity (Fig. 3C, Supplemental Table S2). The last pattern in cluster 5 displays a gradual decline of genes across time. These genes were related to nuclear chromosome and sister chromatin segregation (Fig. 3A, D, Supplemental Table S2).

### Gene pattern and GO analysis revealed effects on DNA damage, G1/S transition phase and chromosome segregation in mPOCs

In mPOCs, we performed the same analysis and selected clusters 1, 2, 3, and 4 among the 6 patterns (Fig. 4A). Genes in cluster 1 displayed a steep upregulation across timepoints (Fig. 4A). Similar to the cluster 3 in cPOCs, GO analysis showed that this pattern was associated to cell death, apoptotic pathway, and DNA damage (Fig. 4B, Supplemental Table S3). Cluster 2 and 3 exhibited opposite trends over time (Fig. 4A). The unique signature in cluster 2 was closely related to G1/S transition of mitotic cell cycle, DNA replication and mRNA transport (Fig. 4C, Supplemental Table S3). On the other hand, genes in cluster 3, associated with histone modification and ubiquitin activity, exhibited an initial decline at 4 h, followed by a sharp upregulation at 24 h post-irradiation (Fig. 4A, Supplemental Table S3). Genes in cluster 4 displayed a gradual downregulation throughout the recovery period post-irradiation (Fig. 4A), and this signature was shown to be cell cycle related, where enriched GO terms included chromosome segregation, mitotic nuclear division and cell cycle phase transition (Fig. 4D, Supplemental Table S3).

### Proteomics analysis revealed disruption in cell adhesion

After assessing the effect of irradiation at the transcriptomic level, we investigated its impact on proteomic profiles. We conducted a proteomic analysis using LC-MS/MS on both cPOCs (*n* = 4) and mPOCs (*n* = 4). Two samples - one in cPOCs at 1 h and one in mPOCs at 4 h post-irradiation - were excluded from the analysis due to the low protein yield after extraction. After removing patient-specific effects, we identified DEPs (FDR < 0.1) by comparing the average expressions of each peptide in the irradiated groups to those of controls. Similar to the transcriptomic data, the irradiated samples at 1 h and 4 h post-irradiation were compared to the 1 h control, and 24 h irradiated samples to the 24 h controls.

**Fig. 5:**
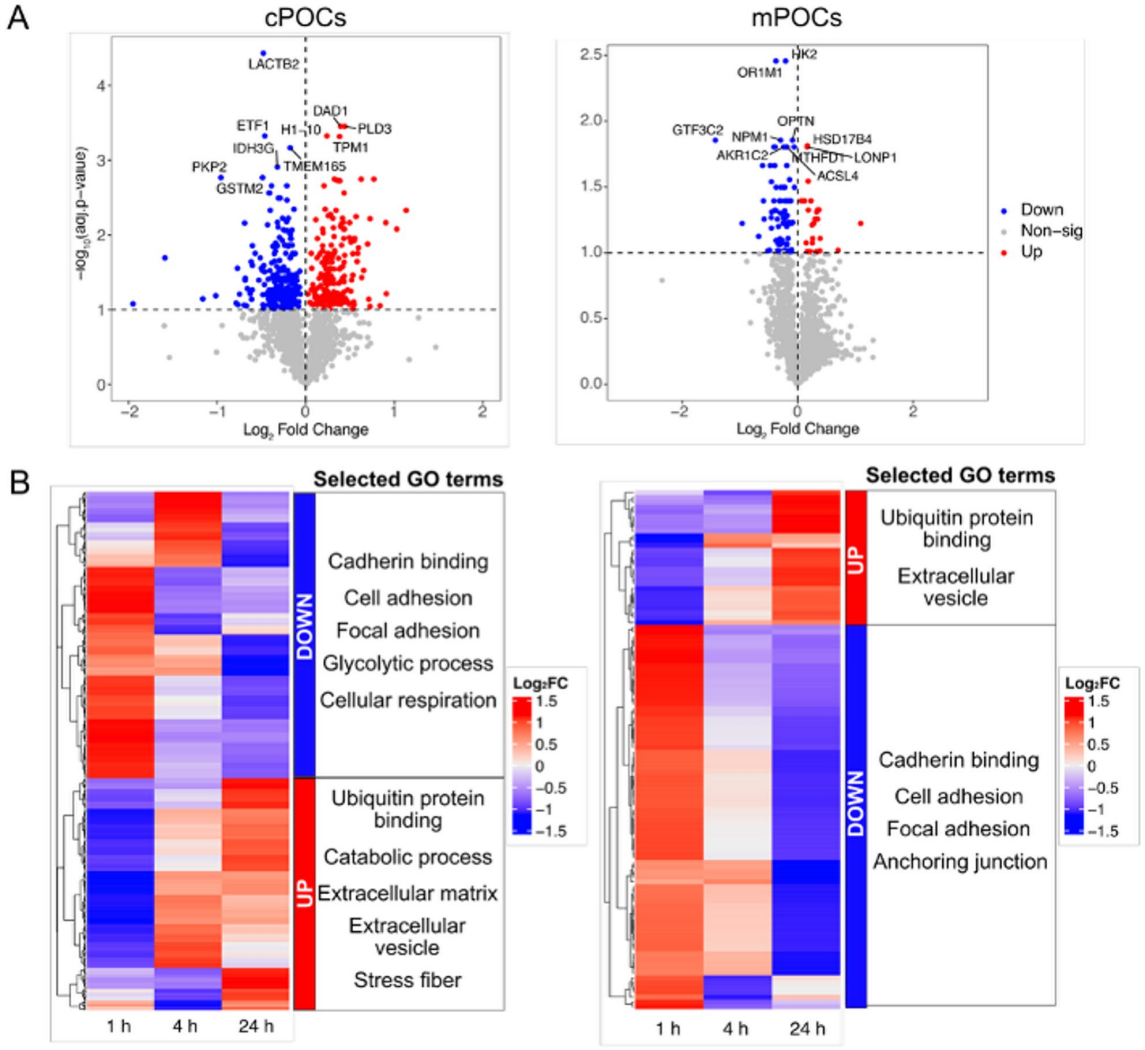
Irradiation induced changes in proteomic profiles of cPOCs and mPOCs. **A** Volcano plots showing the up- and downregulated proteins in cPOCs and mPOCs at 24 h post-irradiation. The top 10 significant DEPs were annotated in the plot. Colour scheme: red indicates protein upregulation, blue indicates protein downregulation, and grey indicates no significant differences in protein expression. **B** Heatmaps show the average log_2_ fold change of DEPs identified in 24 h group across all timepoints and the selected GO terms enriched using the up- and downregulated DEPs in cPOCs and mPOCs. cPOCs, cortex-derived primary ovarian cells; DEPs, differentially expressed proteins; GO, gene ontology; Log_2_FC, log_2_ fold change; mPOCs, medulla-derived primary ovarian cells

In both cPOCs and mPOCs, no significant DEPs were identified at 1 h and 4 h post-irradiation (Supplemental Table S4). On the other hand, 443 DEPs were identified in cPOCs and 110 in mPOCs at 24 h post-irradiation (Supplemental Table S4). Proteins related to cell adhesion (e.g., TPM1, PKP2) and metabolism (e.g., HK2, ACSL4) were found to be highly significant at 24 h in cPOCs and mPOCs, respectively (Fig. 5A). As no DEPs were identified in the early timepoints, we investigated the expression and possible variation of DEPs identified at 24 h post-irradiation also at the 1 h and 4 h timepoints. We plotted the average log_2_ fold change (log_2_FC) of DEPs identified in 24 h group across all timepoints. This allowed us to observe potential patterns in the proteome data, although no statistical significance was identified. As a result, a shift in the protein profile of the selected DEPs was already revealed at earlier timepoints (Fig. 5B). To further explore the affected biological processes, we stratified the DEPs into up- and downregulated groups based on their log_2_FC at 24 h post-irradiation and performed GO over-representation analysis. Consistent with the RNA-seq data, downregulated DEPs were associated with cell adhesion and focal adhesion, while upregulated DEPs were related to ubiquitin-protein binding and extracellular vesicles in both cPOCs and mPOCs (Fig. 5B).

### Irradiation hinders the ability of cPOCs and mPOCs to form Silk-Ovarioids in vitro

**Fig. 6:**
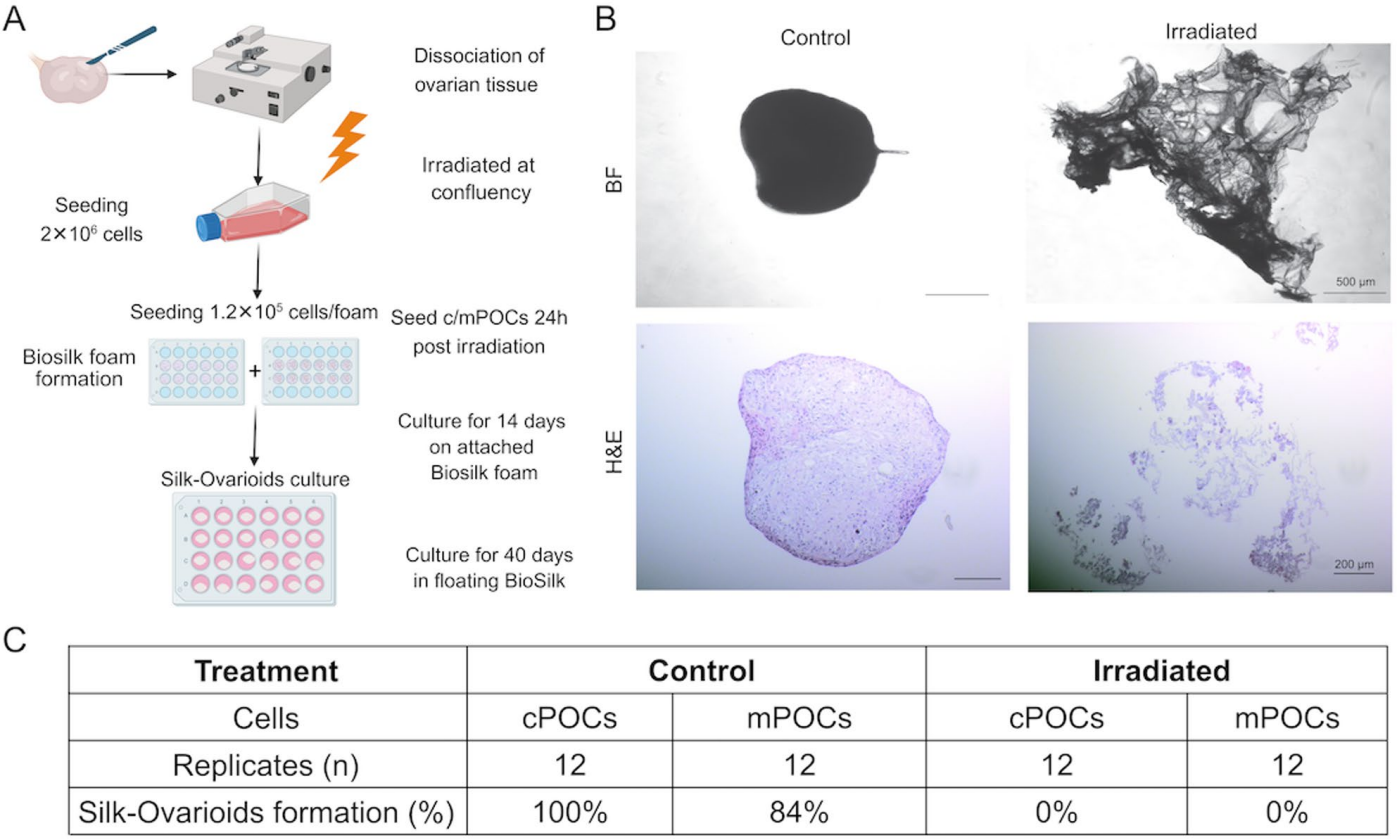
Functional in vitro assessment of Silk-Ovarioid formation in control and irradiated cPOCs and mPOCs. **A** Schematic representation of the experimental approach for Silk-Ovarioids formation (*n* = 2 patients; 6 BioSilk foams/patient). Graph generated using BioRender. **B** Representative brightfield and H&E images of Silk-Ovarioids from control and irradiated POCs after 40 days in free-floating culture. BF scale bar = 500 μm, H&E scale bar = 200 μm. **C** Overview table of the successful Silk-Ovarioids formed from non-irradiated and irradiated cPOCs and mPOCs. BF, bright field; cPOCs, cortex-derived primary ovarian cells; H&E, haematoxylin and eosin; mPOCs, medulla-derived primary ovarian cells

Although no significant cell death was observed throughout the recovery timepoints, we identified transcriptional changes in chromatin segregation, ECM, and cytoskeleton organisation in both cPOCs and mPOCs. These alterations suggest hindered functionality in cell-cell interactions after X-ray exposure. To investigate this, we tested the ability of both cPOCs (*n* = 2) and mPOCs (*n* = 2) to form 3D Silk-Ovarioids in vitro, by seeding cells onto Biosilk scaffolds 24 h post-irradiation, following the in-house protocol [27]. Non-irradiated cPOCs and mPOCs served as control groups for comparison. The cells were cultured on the Biosilk scaffolds for 14 days, after which they were transferred to a floating culture system and observed for up to 40 days (Fig. 6A). For each patient, 6 foams were seeded to form Silk-Ovarioids in non-irradiated and irradiated groups. In the non-irradiated cPOCs group, all 12 foams resulted in Silk-Ovarioids formation (100%), whereas in the irradiated group, only 3 out of 12 foams produced aggregates (25%). Nevertheless, the 3 structures were unstable and disintegrated during the preparation of the samples for histochemical assessment. Similarly, in the non-irradiated mPOCs group, 10 out of 12 foams led to Silk-Ovarioids formation (83%), while none of the irradiated mPOCs formed aggregates throughout the 40 days of culture (0%) (Fig. 6C).

In the non-irradiated controls, well-defined Silk-Ovarioids were observed under an inverted phase-contrast microscope (Fig. 6B). Silk-Ovarioids formed within three days of free-floating culture in both cPOCs and mPOCs controls, respectively. These structures remained stable and well-defined for up to 40 days (Fig. 6B). Haematoxylin and Eosin (H&E) staining further confirmed the correct aggregation in the control group. In fact, Silk-Ovarioids from non-irradiated cPOCs and mPOCs appeared well-defined, containing intact cells within the aggregated structure (Fig. 6B). On the contrary, irradiated cPOCs and mPOCs were hardly visible on the silk fibres and, when present, displayed disorganised cellular architecture (Fig. 6B; Supplemental Fig. S10).

## Discussion

This study aimed, for the first time, to identify the molecular signature of acute high-dose X-ray exposure in human primary ovarian cells isolated from both the cortex (cPOCs) and the medulla (mPOCs). This novel approach contributes to our understanding of radiation-induced ovarian damage and provides groundwork for future studies aimed on developing protective protocols for ovarian tissue in patients undergoing RT.

After screening different doses and time of recovery with KGN granulosa-like cancer cell line, we selected the clinically relevant 10 Gy as the acute high-dose. In the experimental settings outlined, we did not observe significant differences in ATP levels or mitochondrial dehydrogenases activity in irradiated cPOCs and mPOCs. Intracellular ATP levels indicate cellular viability [45], while mitochondrial dehydrogenase activity plays a critical role in regulating cellular proliferation via metabolism [46]. Our findings suggest that a single 10 Gy dose of irradiation can be considered as a sub-lethal dose in our in vitro settings and does not disrupt core cellular processes on the observed timescale, allowing cells to continue being viable and proliferating, comparable to non-irradiated control levels.

Given the absence of changes in ATP levels and mitochondrial dehydrogenase activity following X-ray exposure, we investigated the activation of canonical radiation-induced damage response pathways - i.e., DNA damage, apoptosis, and cell cycle regulation - via immunofluorescence staining of selected markers in our model. DNA damage mechanisms were activated at early timepoints after irradiation, as demonstrated by the upregulation of γ-H2AX and p-Chk1 in both cPOCs and mPOCs. Literature indicates that irradiation-induced DNA damage occurs through single- or double-strand breaks (SSBs or DSBs) or indirectly (~ 70% of DNA damage) by the means of free radicals generated during irradiation [47, 48]. This activates the DNA damage response pathway, although approximately 5% of DSBs remain unrepaired [49], leading to cell cycle arrest to prevent replication of damaged DNA. In response to DNA damage, γ-H2AX is activated at a very early stage [50], and p-Chk1 subsequently initiates the mechanisms of DNA damage repair [51]. In concordance with these data, we observed an early upregulation of p53 pathway, apoptosis, UV response, and mTORC1 signalling at a transcriptomic level at 4 h post-irradiation in both cPOCs and mPOCs. This was further confirmed by the upregulation of DEGs associated with p53-dependent apoptosis in both cPOCs and mPOCs at 4 h according to the gene pattern analysis. Literature describes how the upregulation of p53 pathway is crucial for DNA damage response, leading to either cell cycle arrest or apoptosis [52]. In our experiments, we observed an initial increase in pro-apoptotic markers, namely the active forms of caspase 3 (statistically significant for 1 h for mPOCs) and p53, at the very early timepoint (1 h), followed by a steady reduction at the later culture times (4 and 24 h). This was supported by the overexpression of the anti-apoptotic marker Bcl-2 during the first 4 h post-irradiation, sustaining the hypothesis of the transition from apoptosis to cell cycle blockage. However, these results may be influenced by the unintended selection of surviving cells post-irradiation, a potential bias introduced by the technical procedures of immunostaining assays. Nevertheless, this p53-dependent mechanism has been previously described in immortalized primary retinal pigment epithelium cells, showing a slow recovery of mitosis after 10 Gy irradiation [53].

In mice and human cell lines, it has been reported that radiosensitive tissues display a prolonged p53 signalling activation, up to 7 h after X-ray exposure [54]. This activation has also been widely reported as a marker of radiation sensitivity in oncological patients [54, 55]. In our transcriptomic data, we detected a sustained upregulation of p53 pathway at 24 h post-irradiation, accompanied by overall repression of cell cycle related signals, such as E2F, mTORC1, G2M checkpoint and MYC targets in both cPOCs and mPOCs. Consistent with the mechanism elucidated by Reyes et al. [53] that outlined a p53-dependent cell cycle arrest through p-p21 activation and inhibition of cyclin E, we also identified the lack of p-p53 protein levels and the upregulation of p-p21 in both cPOCs and mPOCs. The mechanism that inhibits the expression of cyclin E seems to affect mPOCs, that display a significant downregulation of this selected cyclin. In addition, the steady downregulation of DEGs throughout the tested timepoints reveals a disruption of chromatin segregation and mitosis, as evidenced by the gene pattern analysis in both cPOCs and mPOCs. Previous reports suggest that effective repair of initial irradiation-induced DNA damage allows cells to continue progressing through the cell cycle. However, if repair is unsuccessful, cells may either undergo cell death and exit the cycle or persist while attempting to resolve genomic instabilities [56]. In addition, analysis at the protein level, cyclin D1remained stable in cPOCs, while mPOCs exhibit decreasing levels of cyclin D1 up to 24 h post-irradiation. This suggests distinct cell cycle responses in cPOCs and mPOCs, further indicating that mPOCs are a more radiosensitive target. These differences in cell-cycle response, together with the higher radiosensitivity of mPOCs relative to cPOCs, are consistent with the elevated proliferative activity of medulla-derived cells, as rapidly cycling populations are generally more radiosensitive [57]. In addition, normoxic culture conditions may have amplified irradiation-induced damage, whereas lower oxygen tensions are known to attenuate it [58]. In our experiments, although both cPOCs and mPOCs showed transcriptomic downregulation of G2/M checkpoints genes, mPOCs showed a significant induction of p-p21 following 10 Gy X-ray exposure, alongside a trend toward higher p-p53, cleaved caspase-3 and BCL2 signals. This suggests a DNA damage response-driven cytostatic response, favouring senescence rather than apoptosis at the examined timepoints [59]. Taken together, these observations indicate that the greater radiosensitivity of mPOCs may derive from their proliferative state combined with a more pronounced and rapid engagement of DNA damage response pathways under normoxic conditions.

One of the main contributors to cell cycle progression is the transcription factor Myc [60]. Specifically, Myc regulates cell cycle progression primarily by suppressing the expression or activity of key cell cycle inhibitors, such as p15, p21 and p27 [61, 62]. Therefore, the downregulation of Myc targets observed in our transcriptomic dataset, together with the upregulation of p-p21 may indicate signs of cell cycle arrest in both cPOCs and mPOCs. Furthermore, *MYC* has been previously reported to impair G1/S checkpoint [9]. Particularly in mPOCs, this mechanism is corroborated by the downregulation of gene patterns involved in the G1/S phase transition at 24 h post-irradiation, suggesting genomic instability [9]. This suggests the downregulation of Myc-dependent pathways represents a primary adaptive response to irradiation in cPOCs, and particularly in mPOCs, reinforcing cell cycle arrest mechanisms and potentially contributing to genomic instability.

Through proteomics analysis, we identified changes in cell adhesion-related proteins post-irradiation. Specifically, irradiation disrupted cell adhesion in cPOCs and mPOCs by downregulating proteins associated with focal adhesion and cadherin binding, which are closely linked to ECM organisation. Particularly, the ECM provides an essential physical support to cells and delivers crucial signals for tissue morphogenesis, differentiation and homeostasis [63]. Among the regulators of ECM-related cell adhesion, integrins and cadherins play key roles in both physiological and pathological conditions [64–66]. It has been reported that the disruption of integrin clusters may reduce adhesion and increase radiosensitivity [67]. On the other hand, the upregulation of N-cadherin in glioblastoma stem cells has been linked to the mediation of adaptive radioresistance [68], suggesting the important role of this protein family in the ECM remodelling and response to RT. Additionally, cell adhesion mechanisms and its regulation are also reported to influence cytoskeleton formation and cell migration [69]. In our study, the disrupted proteome of cell adhesion post-irradiation was confirmed by the inability of irradiated cPOCs and mPOCs to form 3D cellular aggregates, Silk-Ovarioids. Our previous study demonstrated that Silk-Ovarioids derived from both cPOCs and mPOCs can sustain long-term culture, and their formation is strictly dependent on the successful cell-cell interactions and *de novo* ECM formation [27]. Overall, these results highlight the potential damage induced by X-ray exposure, impairing the cellular adhesion mechanisms in long term culture.

The present study expands our understanding of how irradiation impacts POCs derived from both the cortex and the medulla, suggesting that acute high-dose X-ray exposure affects the transcriptomic and proteomic profiles of POCs and disrupts cell adhesion mechanisms. One limitation of this study is the ovarian tissue source. In fact, our samples were retrieved from patients undergoing gender-affirmation surgery who had received androgen treatments prior to surgical removal. Nevertheless, our previous work demonstrated a comparable ovarian cell composition of androgen-exposed ovaries and tissue retrieved from patients undergoing caesarean section, suggesting a limited effect of hormones on the cellular composition of ovarian tissue [15]. However, potential differences related to the functionality of the cells due to the androgen treatments cannot be entirely excluded. Additionally, the impact of X-ray exposure was assessed in a monolayered cell model, limiting the extrapolation of these results to the in vivo ovarian environment. Still, we were able to observe the possible deteriorating effect of RT on the formation of 3D Silk-Ovarioid. Furthermore, this study primarily focused on the somatic cell population essential for ovarian function, and further research is needed to investigate the effects of X-ray exposure on ovarian follicles and their functionality. Despite these limitations, the study has several strengths that enhance its validity and contribution to the field of fertility preservation. Notably, it represents the first study on the effects of irradiation that does not rely on cancer cell lines or animal models, instead utilizes primary cells derived from human ovarian tissue. Moreover, it provides a comprehensive investigation of the impact of irradiation at both the transcriptomic and proteomic levels, followed by functional validation using novel Silk-Ovarioid model.

## Conclusion

In conclusion, this study provides the first novel insights into the response of cPOCs and mPOCs to irradiation. We demonstrate that a single 10 Gy dose is sufficient to induce sublethal damage, primarily affecting cell cycle regulation and cell adhesion. These findings are critical for advancing our understanding of RT-induced ovarian damage and lay the groundwork for future clinically relevant studies aimed at restoring cell adhesion following irradiation, potentially offering a therapeutic target to preserve ovarian function during RT.

## Supplementary Information

Supplementary Material 1.

Supplementary Material 2.

Supplementary Material 3.

Supplementary Material 4.

Supplementary Material 5.

Supplementary Material 6.

Supplementary Material 7.

Supplementary Material 8.

Supplementary Material 9.

Supplementary Material 10.

Supplementary Material 11.

Supplementary Material 12.

Supplementary Material 13.

Supplementary Material 14.

Supplementary Material 15.

## Data Availability

The RNA sequencing count matrix is deposited in Gene Expression Omnibus (GEO) with accession number GSE291604. Raw data is deposited in Swedish National Data Service (SND) with the preliminary link: (https://researchdata.se/en/catalogue/dataset/2025-125/1?previewToken=7373d630-3ab0-4da3-a3a0-7dbaff4f9fee) (reserved DOI: [10.48723/ya3w-an36]). The mass spectrometry proteomics data have been deposited to the ProteomeXchange Consortium via the PRIDE (Perez-Riverol et al., 2022) partner repository with the dataset identifier PXD061796. The code used for the analysis can be found in (https://github.com/tialiv/X-Ovary).

## Abbreviations

RT: Radiotherapy
ECM: Extracellular Matrix
GC: Granulosa Cells
POI: Premature Ovarian Insufficiency
Gy: Gray
POCs: Primary Ovarian Cells
POCs: Cortex derived Primary Ovarian Cells
mPOCs: Medulla derived Primary Ovarian Cells
DPBS: Phosphate Buffered Saline
HAS: Human Serum Albumin
FSD: Focus Skin Distance
DAPI: 4’,6- Diamidino-2-Phenylindole Dihydrochloride
NGI: National Genomics Infrastructure
DEGs: Differentially Expressed Genes
FDR: False Discovery Rate
GSEA: Gene Set Enrichment Analysis
GSVA: Gene Set Variation Analysis
DEPs: Differentially Expressed Proteins
GO: Gene Ontology
p-Chk1: Phosphorylated Chk1 (Ser345)
cl-Cas3: Cleaved Caspase-3
p-p53: Phosphorylated p53 (Ser15)
p-p21: Phosphorylated p21 (Ser146)
DEA: Differential Expression Analysis
H&E: Haematoxylin and Eosin
NES: Normalised enrichment score
PCA: Principal component analysis
