# Supplementary material for: Acute high-dose irradiation disrupts cell adhesion and Silk-Ovarioid formation in human primary ovarian cells
Source: J Ovarian Res. 2026 Jan 2;19:79. doi: 10.1186/s13048-025-01932-8 (PMC12930946; doi:10.1186/s13048-025-01932-8)
Supplement: Supplementary file 5 — Supplementary Material 5. [file 13048_2025_1932_MOESM5_ESM.docx]

# **Acute high-dose irradiation disrupts cell adhesion and Silk-Ovarioid formation in human primary ovarian cells**

Spyridon Panagiotis Deligiannis, *et al*

**Supplemental Material and methods**

## **KGN cell culture**

KGN cells were cultured in DMEM/F12 (Life Technology, USA) supplemented with 10% heat-inactivated foetal bovine serum (HI-FBS; Life Technology, USA), 1% penicillin-streptomycin (Life Technology, USA) and 1% 100X Glutamax^TM^ (Life Technology, USA). Cells were maintained at 37 ^o^C in a 5% CO_2_ humidified atmosphere, with media change every two days. For different experimental set-ups, cells were seeded at different densities, as reported in Table M1.

**Table M1: KGN and primary ovarian cells (POCs) seeding density.** Information of the seeding density for each cell type, divided by experimental approach and plate/flask used.

| **Type of cells** | **Plate/flask** | **Experiment set up** | **Seeding density** |
| --- | --- | --- | --- |
| KGN  cPOCs  mPOCs | 96-well plate | CellTiter and MTT | 1.5 x 10^4^ cells/well |
| KGN  cPOCs  mPOCs | 24-well plate | Immunofluorescence | 1.5 x 10^4^ cells/well |
|  | 96-well plate |  |  |
| cPOCs  mPOCs | 6-well plate | Transcriptomic/proteomics | 1.5 x10^5^ cells/well |
| KGN  cPOCs  mPOCs | T75 flask | Thawing cells | 2 x10^6^ cells/flask |

*Abbreviations:* cPOCs, cortex-derived primary ovarian cells; mPOCs, medulla-derived primary ovarian cells; MTT, (3-[4,5-dimethylthiazol-2-yl]-2,5 diphenyl tetrazolium bromide).

## **Ovarian tissue dissociation and primary ovarian cell culture**

For tissue dissociation, cryopreserved/thawed ovarian cortex and medulla were independently digested into a single-cell suspension using a mechanical and enzymatic method, as previously described (Wagner et al., 2020). Briefly, tissues were finely cut into small pieces using scalpels, and further enzymatically dissociated with 1 mg/ml collagenase IA (Sigma Aldrich, USA), 50 μg/ml Liberase^TM^ (Roche Diagnostics, Germany), and 10 IU/ml DNase I (Roche Diagnostics, Germany) diluted in Dulbecco’s Modified Eagle Medium F12 (DMEM/F12; Life Technology, USA) supplemented with 2.5% HI-FBS (Life Technology, USA). The digestion was carried out in a shaking water bath at 37 ^o^C for 40-50 minutes.

Dissociation was terminated by adding equal volume of DMEM/F12 (Life Technology, USA) supplemented with 10% HI-FBS (Life Technology, USA), followed by centrifugation (300 g, 5 min, centrifuge 5702R; Eppendorf) to collect POCs. Cells were resuspended in Dulbecco’s Modified Eagle Medium, low D-glucose (1 g/L), pyruvate (DMEM low glucose, pyruvate; Gibco, Life Technology, USA), supplemented with 10% HI-FBS (Life Technology, USA), and 1% penicillin-streptomycin (Life Technology, USA). The suspension was filtered through a 40 μm strainer (VWR, USA), and cells (7.5-25 μm diameter) were counted using MOXI mini automated counter (ORFLO Technologies). Cortex-derived primary ovarian cells (cPOCs) and medulla-derived primary ovarian cells (mPOCs) were seeded at densities reported in Table M1, with cultures maintained at 37 ^o^C in a humidified 5% CO_2_ incubator, with media changes every other day.

## **Intracellular ATP level and mitochondrial dehydrogenases activity**

Intracellular ATP levels in KGN (n = 3), cPOCs (n = 8) and mPOCs (n=8) were assessed within 7 days post-irradiation using the CellTiter-Glo® assay (Promega, USA), following manufacturer’s instructions. Briefly, an equal volume of CellTiter-Glo® reagent and medium was mixed on an orbital shaker for cell lysis, incubated for 10 min at room temperature in the dark, and luminescence intensity was measured using a SpectraMax i3x ELISA plate reader (Molecular Devices, Germany).

Irradiation-induced metabolic changes were quantified using the Cell Proliferation Kit I (MTT) (Roche Diagnostics, Germany). Cells were incubated with MTT labelling reagent for 4 h, followed by overnight incubation with solubilization buffer. Absorbance was measured at 550 nm wavelength with a reference wavelength >650 nm, using the SpectraMax i3x ELISA plate reader (Molecular Devices).

## **Immunofluorescence staining**

Irradiated KGN (n=1), cPOCs (n=3) and mPOCs (n=3) were fixed with 4% methanol-free formaldehyde (FA; Sigma-Aldrich) at 1 h, 4 h, and 24 h post-irradiation for immunofluorescence staining. Non-irradiated controls were cultured and fixed alongside all the timepoints (KGN) and the 1 h irradiated samples (cPOCs and mPOCs). KGN cells were detached, fixed, and sedimented to a SuperFrost® Plus slides (Fisher Scientific, USA) using a Cytospin 4 centrifuge (Thermo Scientific, USA) before the staining process. Cells were permeabilised using a blocking buffer containing DPBS (Life Technologies, Paisley, UK), 0.3% Triton X-100 (Sigma-Aldrich), 3% normal donkey serum (NDS; Nordic Biosite) and 5% bovine serum albumin (BSA; Sigma-Aldrich). Primary antibody incubation (Table 2) was performed overnight at 4 ^o^C on an orbital shaker, diluted in antibody buffer containing DPBS (Life Technologies, Paisley, UK) and 1% NDS (Nordic Biosite). Negative controls were incubated with blocking buffer.

**Table M2:** Overview of antibodies used for immunofluorescence staining.

| **Type of marker** | **Antibody** | **Host** | **Company** | **Catalog Number** | **Dilution** |
| --- | --- | --- | --- | --- | --- |
| **DNA Damage** | p-Histone H2AX (Ser139) | Mouse | Cell Signaling Technology | 80312 | 1: 200 |
|  | p-Chk1 (Ser345) | Rabbit |  | 2348 | 1: 1000 |
| **Apoptosis** | p-p53 (Ser15) | Mouse | Invitrogen | MA5-15229 | 1: 200 |
|  | Cleaved caspase 3 | Rabbit | Cell Signaling Technology | 9661 | 1: 400 |
|  | Bcl-2 | Mouse | Santa Cruz Biotechnology | sc-7382 | 1: 50 |
| **Cell cycle** | Cyclin D1 | Rabbit | Cell Signaling Technology | 555065 | 1: 1000 |
|  | Cyclin E | Mouse | Invitrogen | 32-1500 | 1: 250 |
|  | p-p21 (Ser146) | Mouse | Santa Cruz Biotechnology | sc-377515 | 1: 250 |

After washing in washing buffer (PBST) containing DPBS (Life Technologies, Paisley, UK) and 0.3% Triton X-100 (Sigma-Aldrich), cells were incubated for 1 h with secondary antibody (Table 3). When primary antibodies from the same host species were used within the same sample, a double-blocking procedure was performed to prevent cross-reactivity. Briefly, at the end of secondary antibody incubation, a second blocking step was performed before overnight incubation with the same-host primary antibody. 4’,6-diamidino-2-phenylindole dihydrochloride (DAPI; Thermo Fischer Scientific) was used for nuclear counterstain (1:1000 dilution in antibody buffer).

**Table M3:** Overview on secondary antibody used for immunofluorescence staining.

| **Primary antibody used** | **Secondary Antibody** | **Host** | **Company** | **Dilution** |
| --- | --- | --- | --- | --- |
| **p-Histone H2AX (Ser139)** | Anti-mouse Alexa Fluor 647 | Donkey | Invitrogen | 1:200 |
| **p-Chk1 (Ser345)** | Anti-rabbit Alexa Fluor 594 |  | Santa Cruz Biotechnology |  |
| **p-p53 (Ser15)** | Anti-mouse Alexa Fluor 647 |  | Invitrogen |  |
| **Cleaved caspase 3** | Anti-rabbit Alexa Fluor 594 |  | Santa Cruz Biotechnology |  |
| **Bcl-2** | Anti-mouse Alexa Fluor 488 |  | Invitrogen |  |
| **Cyclin D1** | Anti-mouse Alexa Fluor 647 |  | Invitrogen |  |
| **Cyclin E** | Anti-rabbit Alexa Fluor 594 |  | Santa Cruz Biotechnology |  |
| **p-p21 (Ser146)** | Anti-mouse Alexa Fluor 488 |  | Invitrogen |  |

Imaging was performed using a 20x air objective with 1.5x lens on a Nikon Eclipse Ti2 confocal microscope, with emission filters set for blue (438/24), green (511/20), red (560/25), and far-red (685/40) detection using Kinetix sCMOS camera (Crest Optics). Image processing was carried out in Omero figure software, with contrast and brightness adjusted uniformly per panel.

For immunofluorescence quantification, CellProfiler (version 4.2.5) was used to create image analysis pipelines for immunofluorescence image quantification (Stirling et al., 2021). Briefly, the images were corrected for non-homogeneous illumination by applying Gaussian filtered for DAPI signal. Individual cells were identified by nuclear fluorescence, and fluorescence intensity per marker was measured and compiled in RStudio. Protein expression was visualised as fluorescence intensity ratios.

## **RNA extraction and library preparation**

RNA extraction was performed from cPOCs (n = 5) and mPOCs (n = 5) using the RNeasy Micro Kit (Qiagen, Germany) following the manufacturer’s instructions. Cells were lysed in 75 μL of Buffer RLT (Qiagen, Germany), and genomic DNA contamination was removed with DNase I (Qiagen, Germany) treatment. RNA was eluted with 14 μL of RNase-free water and concentration was measured using a NanoPhotometer (IMPLEN, Nordic Biolabs). RNA quality was assessed through the Agilent Bioanalyzer 2,000 (Agilent, USA) and only samples with RNA integrity value (RIN) > 9 and A260/A280 > 1.8 were used for library construction. Libraries were prepared using the Illumina Stranded mRNA Prep Ligation protocol (Illumina, USA) with 10 ng of RNA input.

## **Proteomics sample preparation**

Irradiated cPOCs (n = 4) and mPOCs (n = 4), along with their respective controls, were snap-frozen and thawed on ice and lysed with 10 µL of 8 M urea, followed by sonication (5 min) in water bath. Next, 70 µL of 0.5 M NaCl in 50 mM Tris-HCl (pH 8.5) and 0.8 µL 100x of protease inhibitor (Roche Diagnostic, Germany) were added. After an additional 5 min of sonication, lysates were centrifuged at 13,000 *g* for 10 min at 4 °C and protein concentration was measured using BCA assay (Pierce, USA).

A lysate volume corresponding to 7.7 µg of protein was supplemented with 1 M urea and 438 mM NaCl in Tris-HCl buffer to final volume of 75 µL. Proteins were reduced with 2.8 µL of 250 mM dithiothreitol (Sigma-Aldrich, USA) and incubated at 37 °C for 45 min while shaking at 400 rpm on a block heater. Alkylation was performed with 3.1 µL of 500 mM iodoacetamide (Sigma-Aldrich, USA), followed by 30 min incubation at room temperature while shacking at 400 rpm in the dark. Proteolytic digestion was carried out with 0.4 µg sequencing grade modified trypsin (Promega, USA) and incubated for 16 h at 37 °C. The digestion was terminated with 4.5 µL of concentrated formic acid (FA) and incubating at room temperature (5 min). Samples were cleaned using a C18 Hypersep plate with 40 µL bed volume (Thermo Fisher Scientific, USA) and dried in a vacuum concentrator (Eppendorf, Germany).

Samples were labelled with TMT-10plex (Thermo Fisher Scientific, USA) isobaric reagents. Peptides were solubilized in 70 µL of 50 mM triethylammonium bicarbonate and mixed with 100 µg TMT-10plex reagents in anhydrous acetonitrile (ACN) and incubated at room temperature for 2 h. Unreacted reagents were quenched with 6 µL of hydroxyamine at room temperature for 15 min. Samples were then combined, dried in vacuum and cleaned on C18 Hypersep plate.

### **Liquid chromatography-tandem mass spectrometry**

The TMT-10plex labelled peptide samples were reconstituted in solvent A, and approximately 2 µg of the samples were injected onto a 50 cm long EASY-Spray C18 column (Thermo Fisher Scientific) connected to an Ultimate 3000 nanoUPLC system (Thermo Fisher Scientific). A 90-minute gradient was used: 4-26% solvent B (98% ACN, 0.1% FA) for 90 min, 26-95% for 5 min, and 95% solvent B for 5 min, with a flow rate of 300 nL/min. Mass spectra were acquired using a Q Exactive HF hybrid quadrupole-Orbitrap mass spectrometer (Thermo Fisher Scientific), scanning from *m/z* 375 to 1700 at a resolution of R = 120,000 (at *m/z* 200), targeting 1x10^6^ ions for a maximum injection time of 80 ms. This was followed by data-dependent higher-energy collisional dissociation (HCD) fragmentation of the top 18 precursor ions with charge states from 2+ to 7+, using a 45-s dynamic exclusion. Tandem mass spectra were acquired with a resolution of R = 60,000, targeting 2x10^5^ ions for a maximum injection time of 54 ms, with quadrupole isolation width set to 1.4 Th and normalized collision energy set to 34%.

## **Formation and culture of Silk-Ovarioids**

Monolayer cultures of cPOCs (n = 2) and mPOCs (n = 2) were detached with TrypLE (Thermo Fisher Scientific) 24 h post-irradiation and utilised for Silk-Ovarioids formation, as previously described (Di Nisio et al., 2024). Briefly, 20 μl drops of Biosilk^TM^ were pipetted into 24-well plate and air bubbles were introduced to create dense foams. Irradiated and non-irradiated (control) cPOCs and mPOCs were seeded onto the foams (density: 1.2 x 10^5^ cells/foam). The foams were incubated at 37 ^o^C for 20 min to allow stabilisation, then overlaid with 700 μl of culture media and incubated at 37 ^o^C with 5% CO_2_ for 14 days, with daily media changes.

On day 14, the foams were detached using a spatula, divided into two equal portions, and transferred into a flat-bottom ultra-low attachment 24-well plate (Corning, USA) for further free-floating culture. Silk-Ovarioids were cultured for 40 more days with daily media changes. At the end of the culture period, they were fixed in 4% FA for morphological evaluation.

**Supplemental Figures and figure legends**

**Supplemental Figure S1.** **Intracellular ATP levels and mitochondrial activity in KGN cells following 2, 4 and 10 Gy irradiation.** Bar graph shows (A) relative ATP luminescence production and (B) normalized absorbance reflecting mitochondrial activity at 4 h, 24 h, 48 h, 72 h, 120 h and 168 h post-irradiation after a single dose of 2, 4, and 10 Gy of irradiation. Data are presented as mean ± SEM. Statistical analysis was performed using one-way ANOVA. a.u., arbitrary units; Gy, Gray.


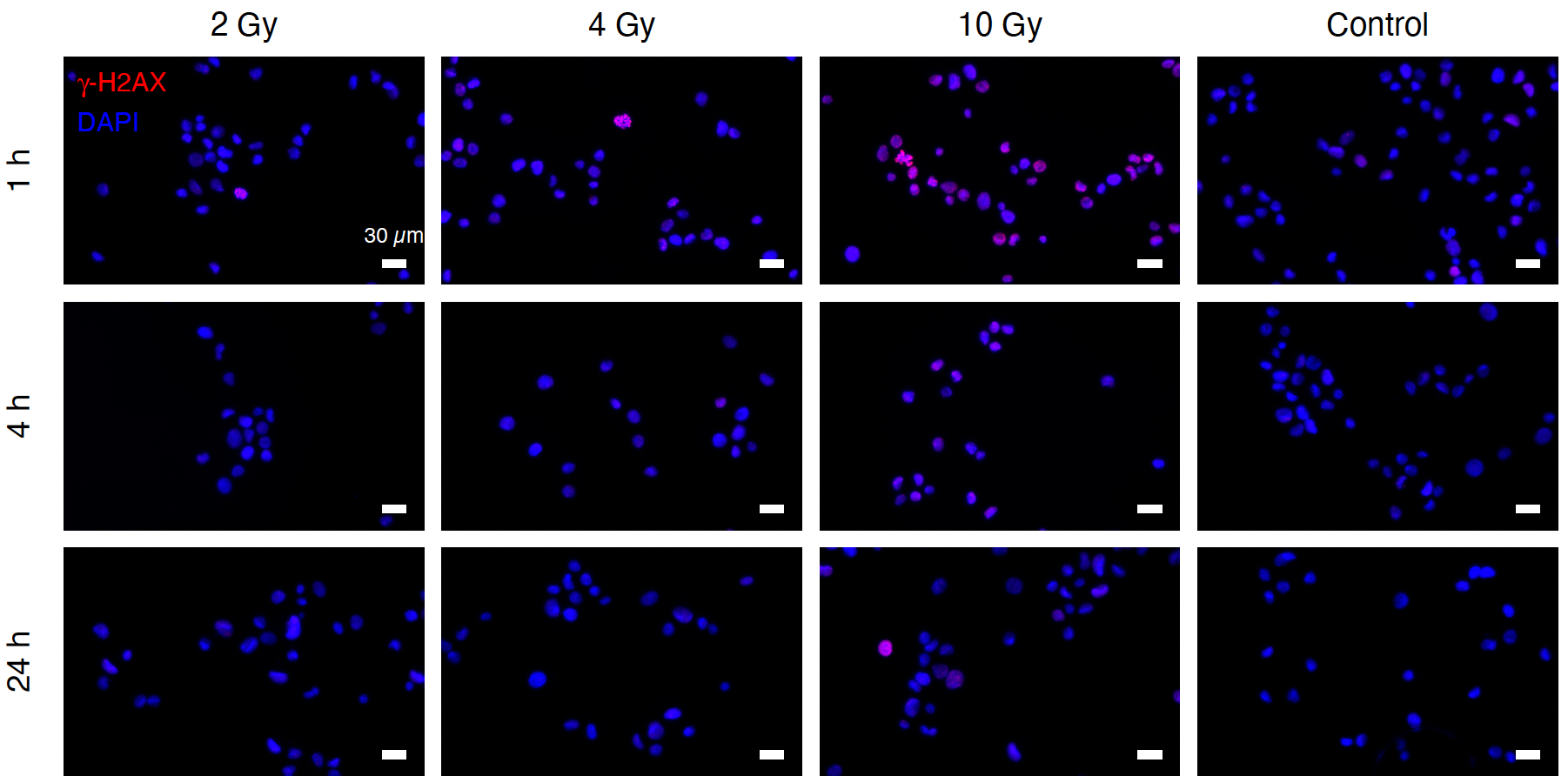


**Supplemental Figure S2. DNA damage in KGN following 2, 4 and 10 Gy irradiation.** Representative images in irradiated and control samples. The cells were immunolabeled with DNA damage-related marker: γ-H2AX. Scale bars represent 30 μm. Gy, Gray.

**Supplemental Figure S3. Intracellular ATP levels and mitochondrial activity in cPOCs and mPOCs following 10 Gy irradiation.** Bar graphs show (A) relative ATP luminescence production and (B) normalized absorbance reflecting mitochondrial activity at 4 h, 24 h, 72 h (short-term culture), 120 h and 168 h (long-term culture) after a single dose of 10 Gy of irradiation with controls at 4 h and 120 h (grey bars). Data are presented as mean ± SEM. Statistical analysis was performed using one-way ANOVA. a.u., arbitrary units; cPOCs, cortex-derived primary ovarian cells; Gy, Gray; mPOCs, medulla-derived primary ovarian cells.


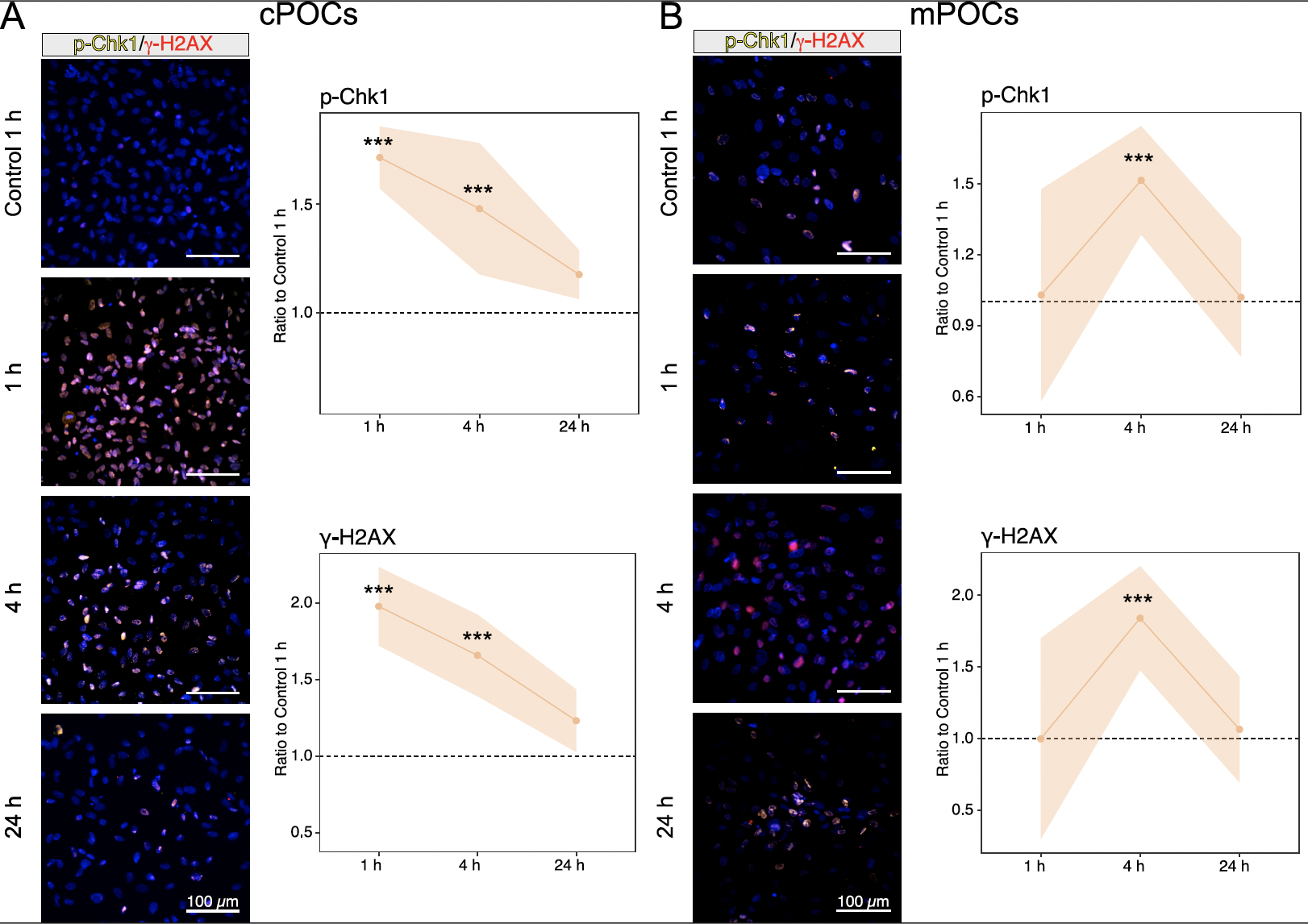


**Supplemental Figure S4. Expression of DNA damage related markers in cPOCs and mPOCs.** Representative images and ratio of fluorescence intensity in irradiated samples compared to the 1 h control of (A) cPOCs and (B) mPOCs. The cells were immunolabeled with DNA damage-related markers: γ-H2AX (red) and p-Chk1 (yellow). Scale bars represent 100 μm. The ratio of mean fluorescent intensity value is shown, with the shaded area around the dots indicating the standard deviation. The ratios were calculated by dividing the mean fluorescence intensity of each irradiated sample by that of the control. Statistical significance was analysed using Kruskal-Wallis test with Dunn’s correction and is displayed by asterisks (*). *** p < 0.001. cPOCs, cortex-derived primary ovarian cells; mPOCs, medulla-derived primary ovarian cells.

**Supplemental Figure S5. Expression of apoptosis-related markers in cPOCs and mPOCs.** Representative images and ratio of fluorescence intensity in irradiated samples compared to the 1 h control of (A) cPOCs and (B) mPOCs. The cells were immunolabeled with apoptosis-related markers p-p53 (red), Bcl-2 (green), and cl-Cas 3 (yellow). Scale bars represent100 μm. The ratio of mean fluorescence intensity value is shown, with the shaded area around the dots indicating the standard deviation. The ratios were calculated by dividing the mean fluorescence intensity of each irradiated sample by that of the control. Statistical significance was analysed using Kruskal-Wallis test with Dunn’s correction and is indicated by asterisks (*). * p < 0.05; ** p < 0.01; *** p < 0.001. cPOCs, cortex-derived primary ovarian cells; mPOCs, medulla-derived primary ovarian cells.

**Supplemental Figure S6.** **Expression of cell cycle-related markers in cPOCs and mPOCs.** Representative image and ratio of fluorescence intensity in irradiated samples compared to the 1 h control of (A) cPOCs and (B) mPOCs. The cells were immunolabeled with cell cycle-related markers cyclin D1 (yellow), cyclin E (red), and p-p21 (green). Scale bars represent 100 μm. The ratio of mean fluorescence intensity value is shown, with the shaded area around the dots indicating the standard deviation. The ratios were calculated by dividing the mean fluorescence intensity of each irradiated sample by that of the control. Statistical significance was analysed using Kruskal-Wallis test with Dunn’s correction and is indicated by asterisks (*). * p < 0.05; ** p < 0.01; *** p < 0.001. cPOCs, cortex-derived primary ovarian cells; mPOCs, medulla-derived primary ovarian cells.

**Supplemental Figure S7. Transcriptomic changes in the non-irradiated control cPOCs and mPOCs.** PCA of top 500 highly variable genes after removing batch effect and significant enriched hallmark gene sets predicted by GSEA using DEGs identified in 24 h control vs 1 h control comparison in (A) cPOCs (B) and mPOCs. NES is shown on the x axis. The size of dots represents –log_10_(adjusted p-value). cPOCs, cortex-derived primary ovarian cells; DEGs, differentially expressed genes; GSEA, gene set enrichment analysis; mPOCs, medulla-derived primary ovarian cells; NES, normalised enrichment score; PCA, principal component analysis.

**Supplementary Figure S8.** **Overall transcriptomic changes induced by irradiation.** PCA of top 500 highly variable genes after removing batch effect comparing 1 h and 4 h post-irradiation with 1 h control and 24 h post-irradiation with 24 h control, (A) in cPOCs and (B) in mPOCs. Two outliers were removed from cPOCs dataset in 24 h post-irradiation groups. (C) GSEA results using all expressed genes ranked by log_2_ fold change against hallmark gene set at 1 h and 4 h time points in cPOCs and mPOCs. NES is shown on the x axis. Colors of bars indicate adjusted p-values. cPOCs, cortex-derived primary ovarian cells; GSEA, gene set enrichment analysis; mPOCs, medulla-derived primary ovarian cells; NES, normalised enrichment score; PCA, principal component analysis.


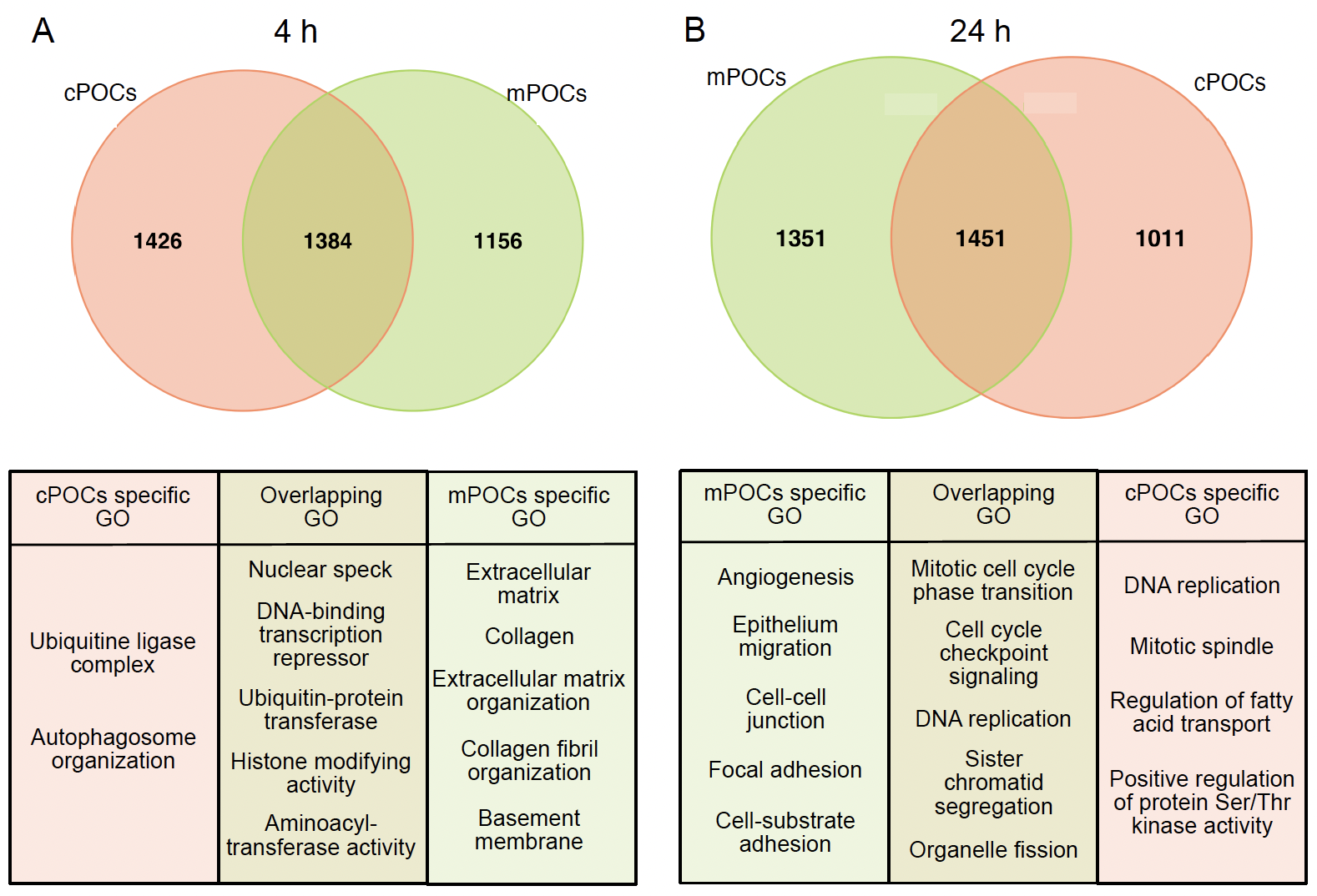


**Supplemental Figure S9.** **Common and group-specific effects between cPOCs and mPOCs induced by irradiation.** Venn diagram shows the number of overlapping and group-specific DEGs between cPOCs and mPOCs, and the table reports the selected significantly enriched GOs for these DEGs (A) at 4 h and (B) 24 h post-irradiation. cPOCs, cortex-derived primary ovarian cells; mPOCs, medulla-derived primary ovarian cells; GO, gene ontology.

**Supplemental Figure S10. Representative images of Silk-Ovarioids derived from irradiated and non-irradiated cPOCs and mPOCs.** Brightfield and H&E images of Silk-Ovarioids derived from irradiated and non-irradiated cPOCs and mPOCs after 40 days in free-floating culture. BF images: scale bar=500 μm; H&E images: scale bar=200 μm. BF, brightfield; cPOCs, cortex-derived primary ovarian cells; H&E, haematoxylin and eosin; mPOCs medulla-derived primary ovarian cells.

**Supplemental Tables legends**

**Supplemental Table S1:** Upregulated (positive binary logarithm of fold change (log2FoldChange)) and downregulated (negative log2FoldChange) DEGs in irradiated (IR) 1 and 4 h *vs* 1 h control cPOCs/mPOCs and IR 24 h *vs* 24 h control cPOCs/mPOCs.

**Supplemental** **Table S2:** Gene pattern enriched GO against all annotated genes in cPOCs.

**Supplemental** **Table S3:** Gene pattern enriched GO against all annotated genes in mPOCs.

**Supplemental Table S4:** Upregulated (positive log2FoldChange) and downregulated (negative log2FoldChange) DEPs in irradiated (IR) 24 h cPOCs *vs* 24 h control cPOCs/mPOCs.
