# Supplementary figures and images for: Acute high-dose irradiation disrupts cell adhesion and Silk-Ovarioid formation in human primary ovarian cells
Source: J Ovarian Res. 2026 Jan 2;19:79. doi: 10.1186/s13048-025-01932-8 (PMC12930946; doi:10.1186/s13048-025-01932-8)

# ATP production in KGN cells

A

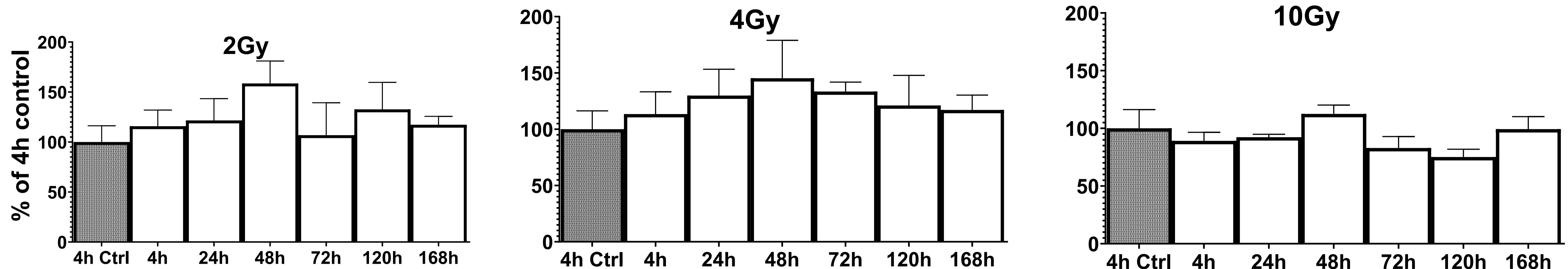

# Mitochondrial activity in KGN cells

B

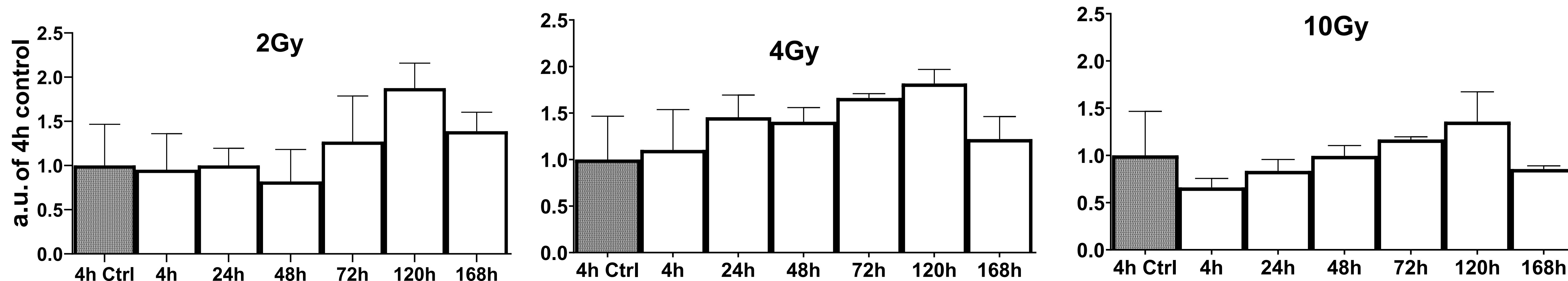

Supplement: Supplementary file 6 — Supplementary Material 6. [file 13048_2025_1932_MOESM6_ESM.pdf]

A

cPOCs

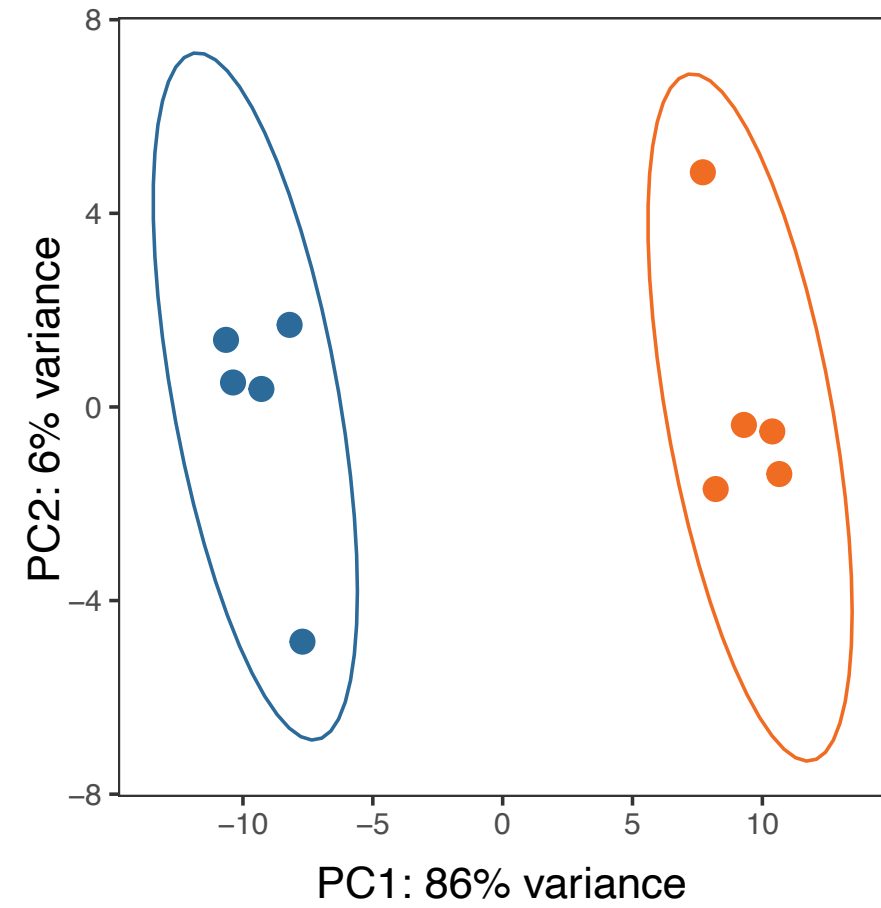

● Control 1 h    ● Control 24 h

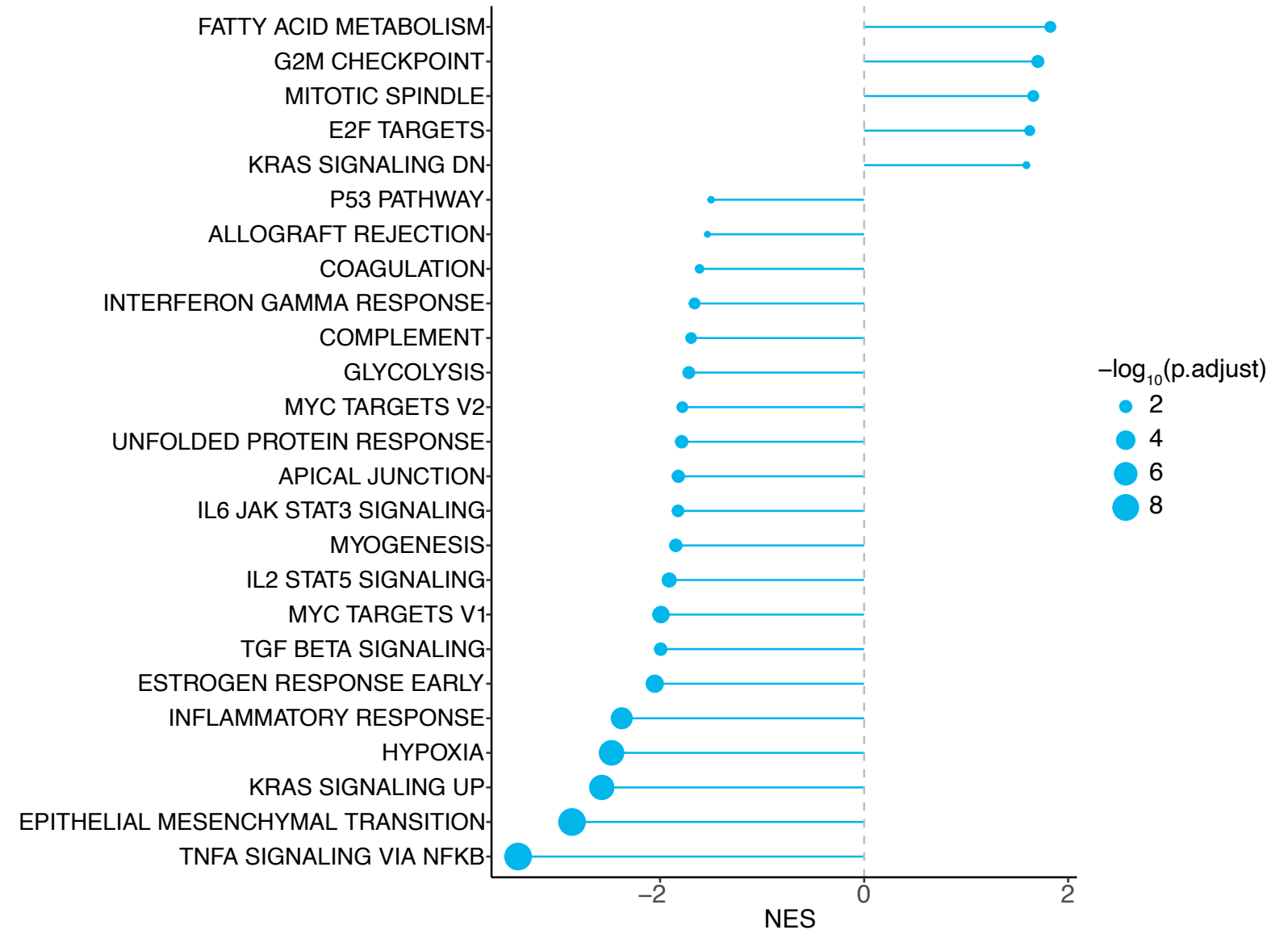

B

mPOCs

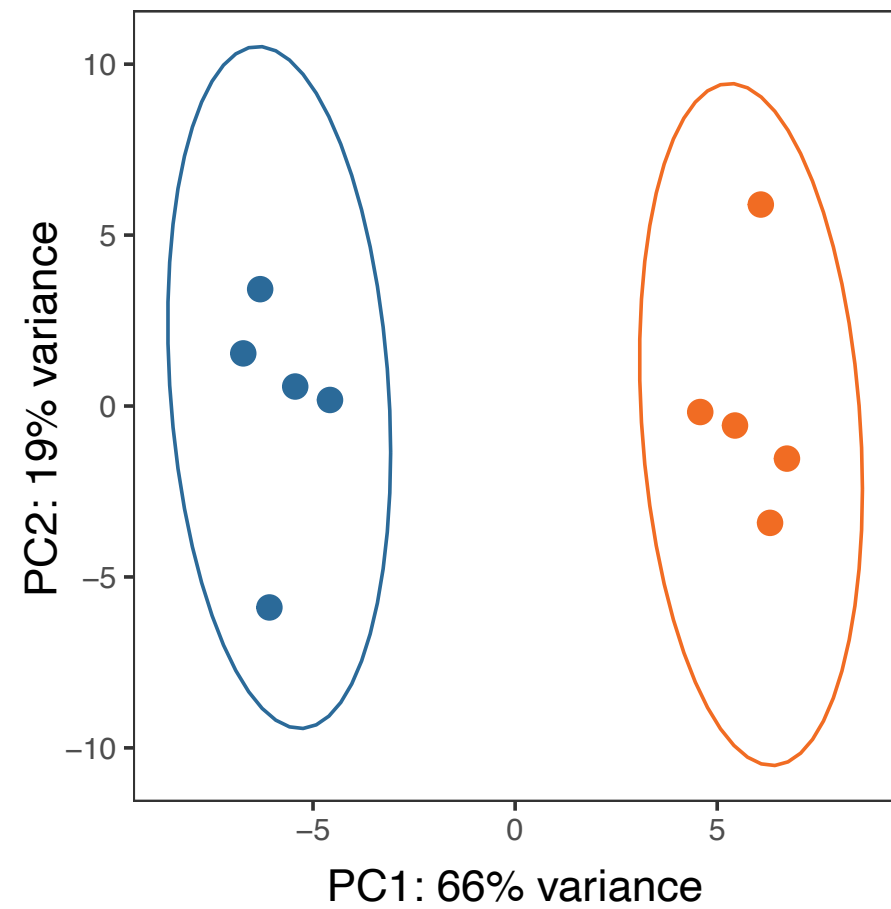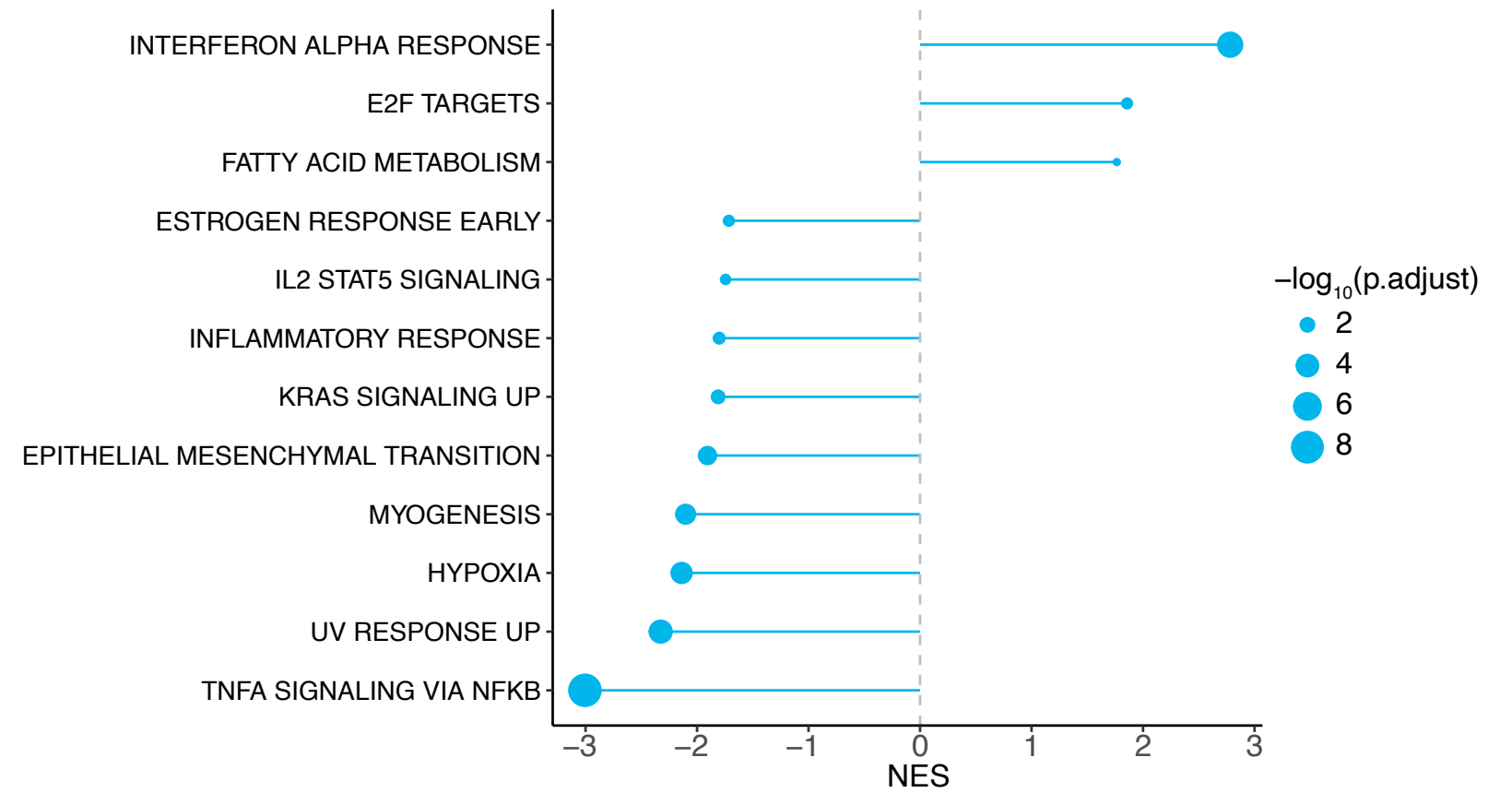

Supplement: Supplementary file 7 — Supplementary Material 7. [file 13048_2025_1932_MOESM7_ESM.pdf]

A

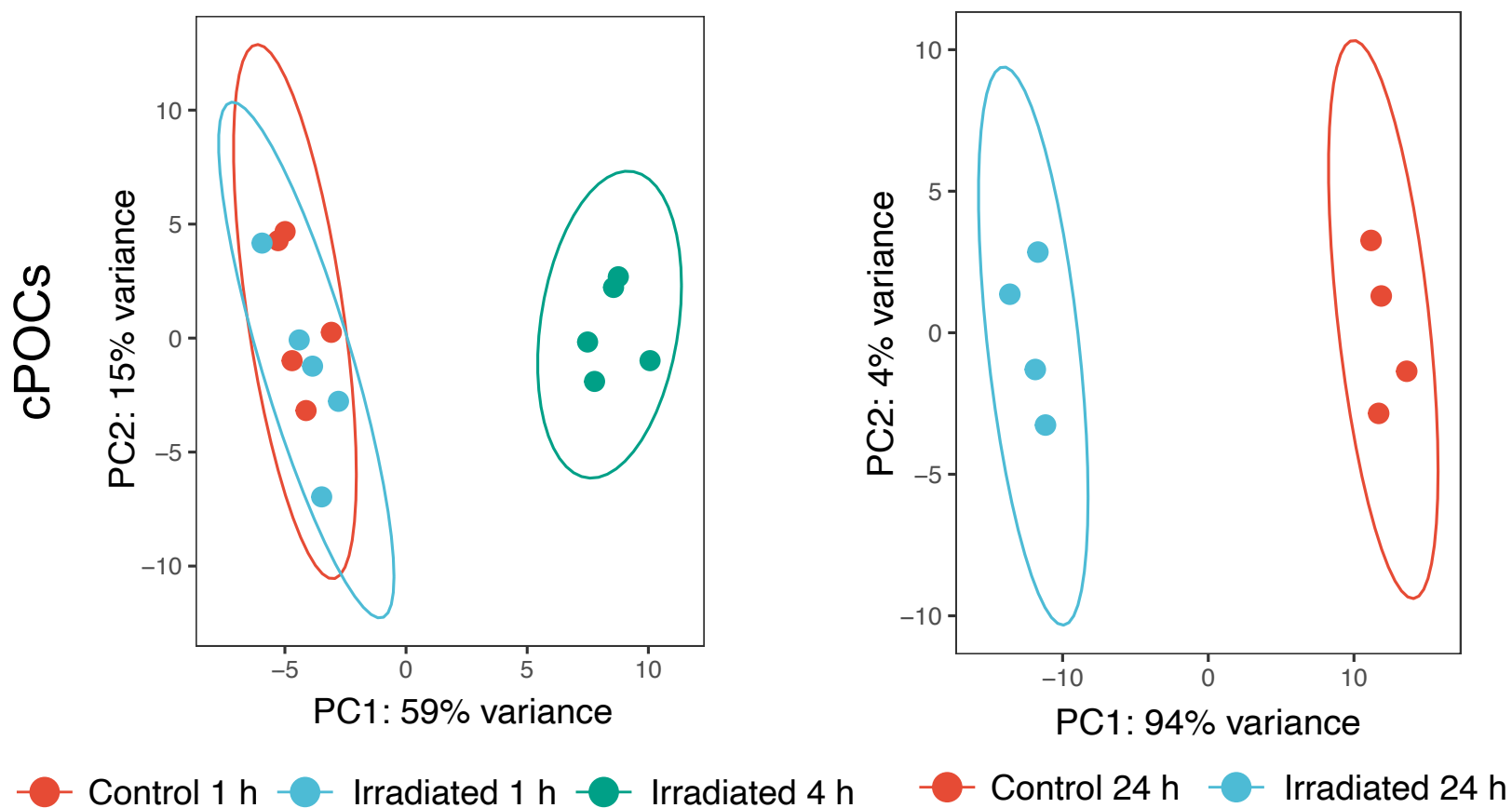

B

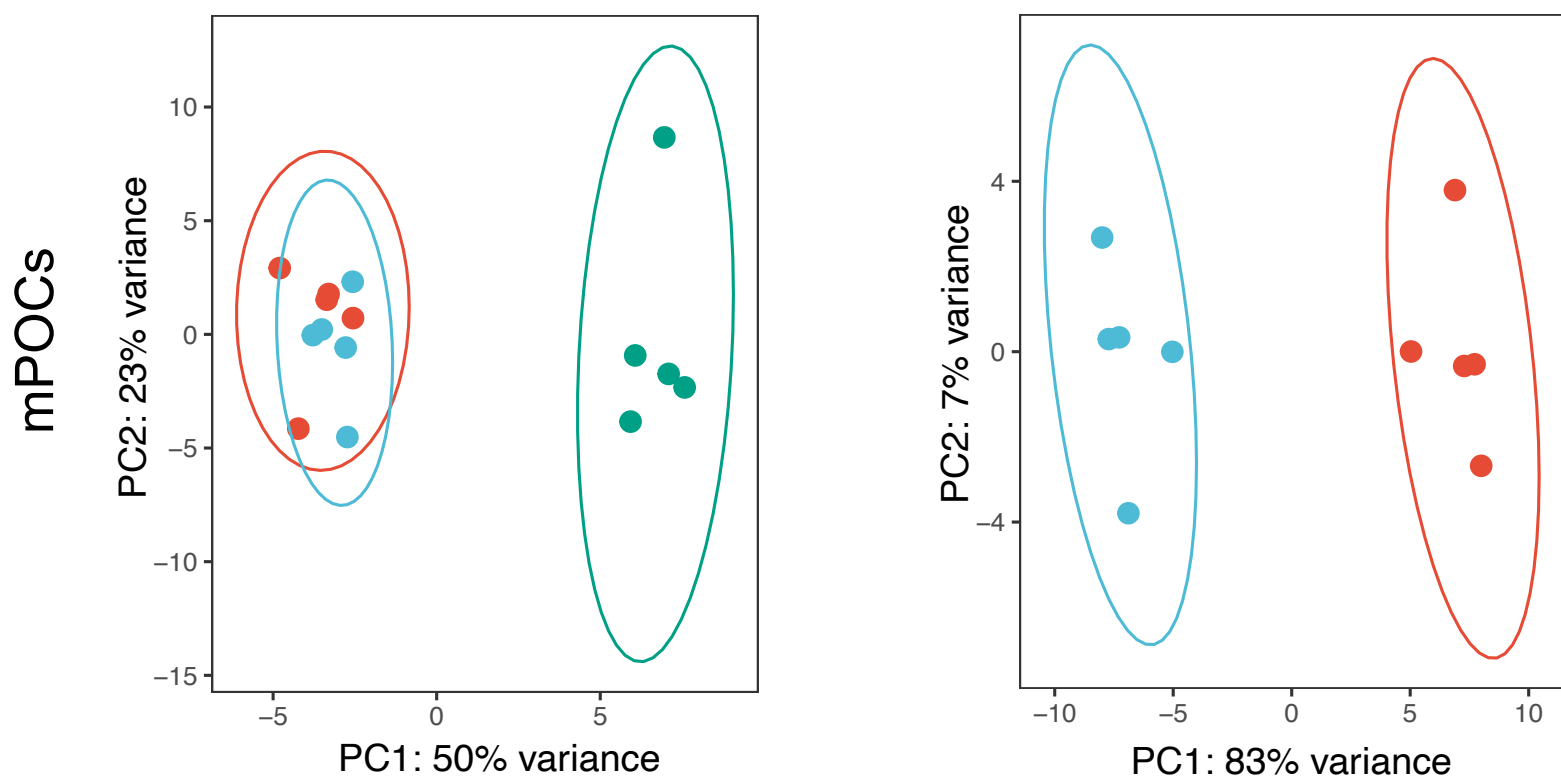

C

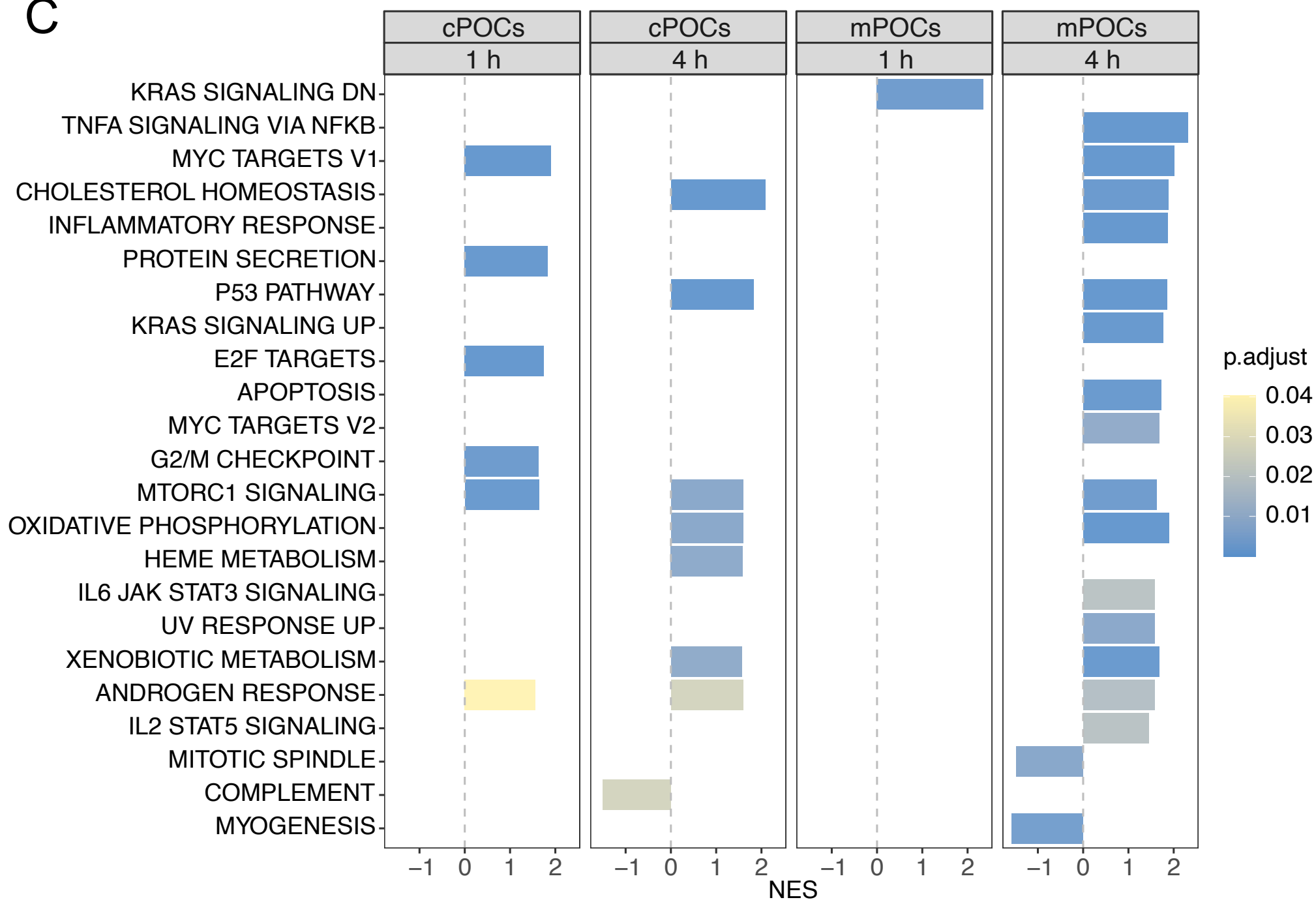

Supplement: Supplementary file 8 — Supplementary Material 8. [file 13048_2025_1932_MOESM8_ESM.pdf]

# ATP production in cPOCs and mPOCs

**A**

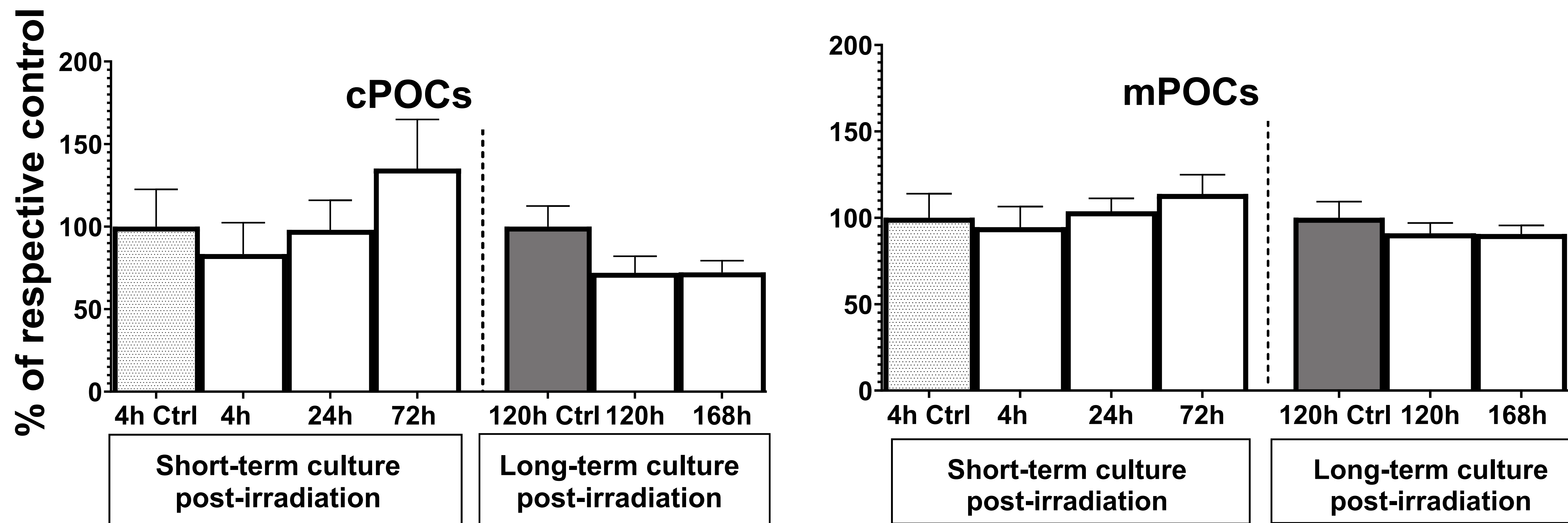

## Mitochondrial activity in cPOCs and mPOCs

**B**

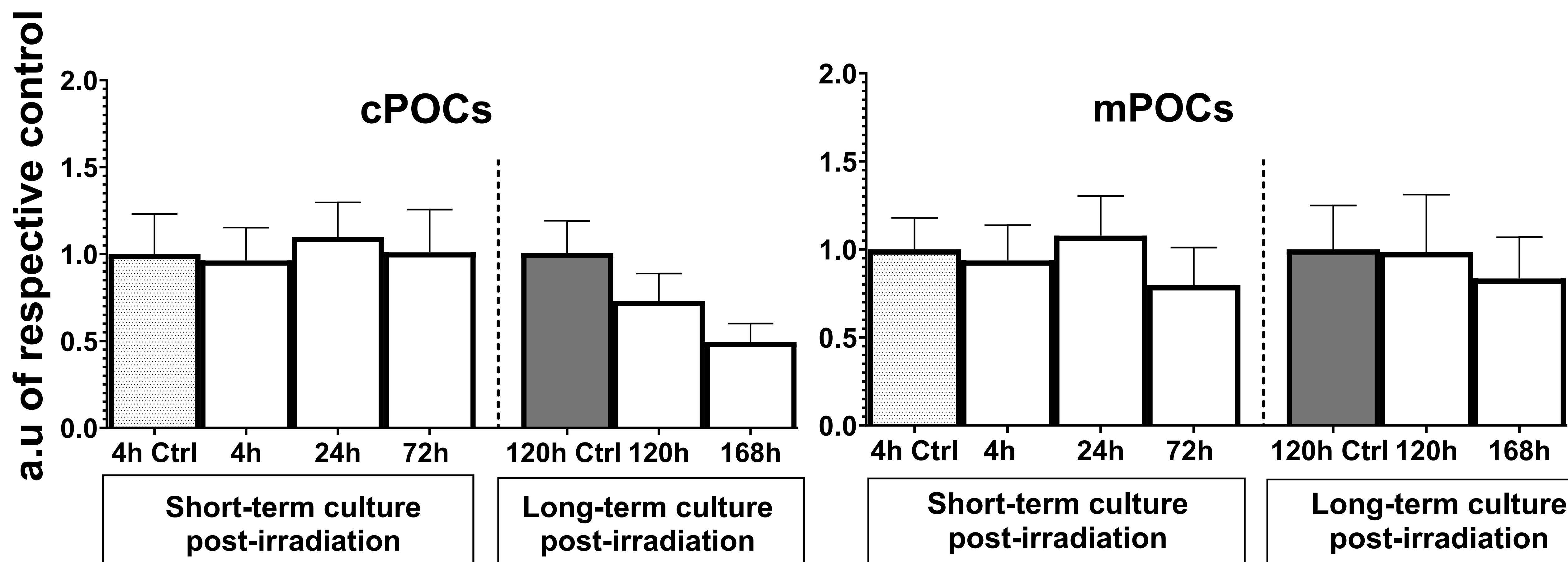

Supplement: Supplementary file 9 — Supplementary Material 9. [file 13048_2025_1932_MOESM9_ESM.pdf]

A

cPOCs

Control 1 h

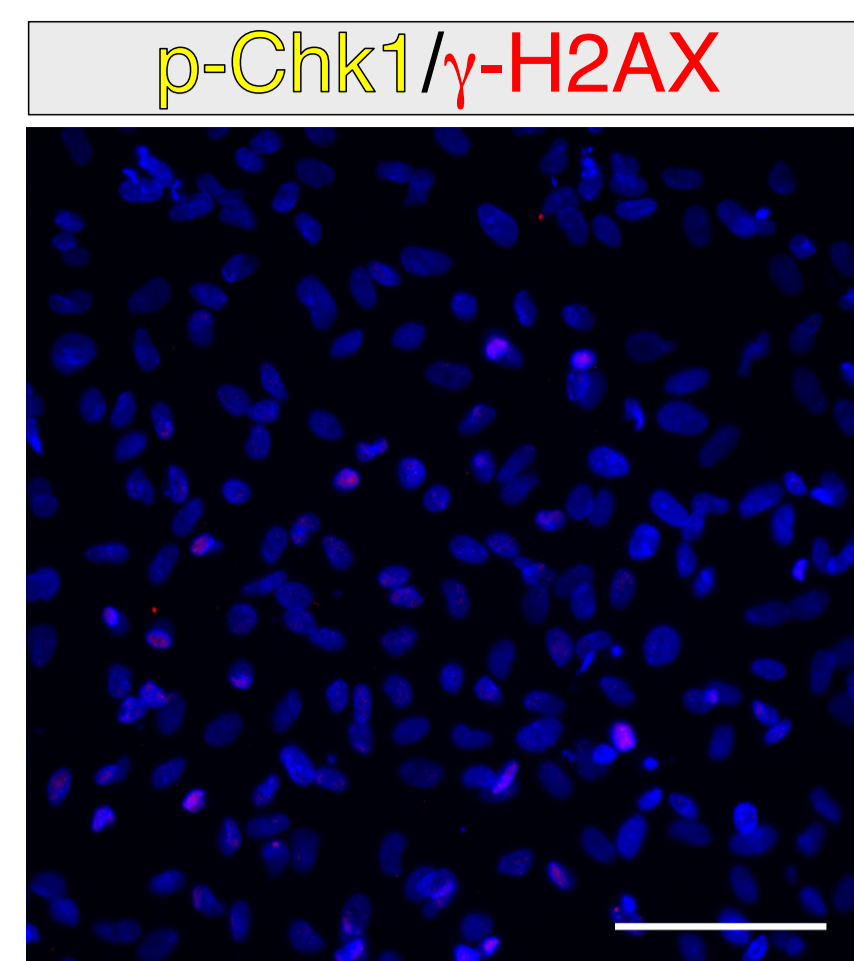

1 h

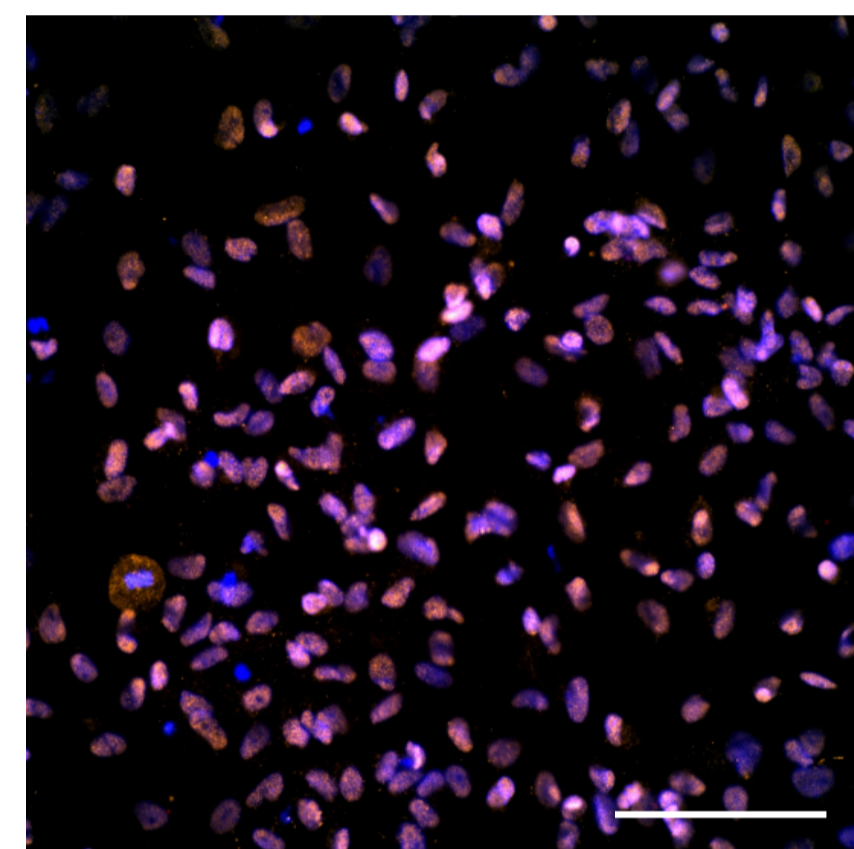

4 h

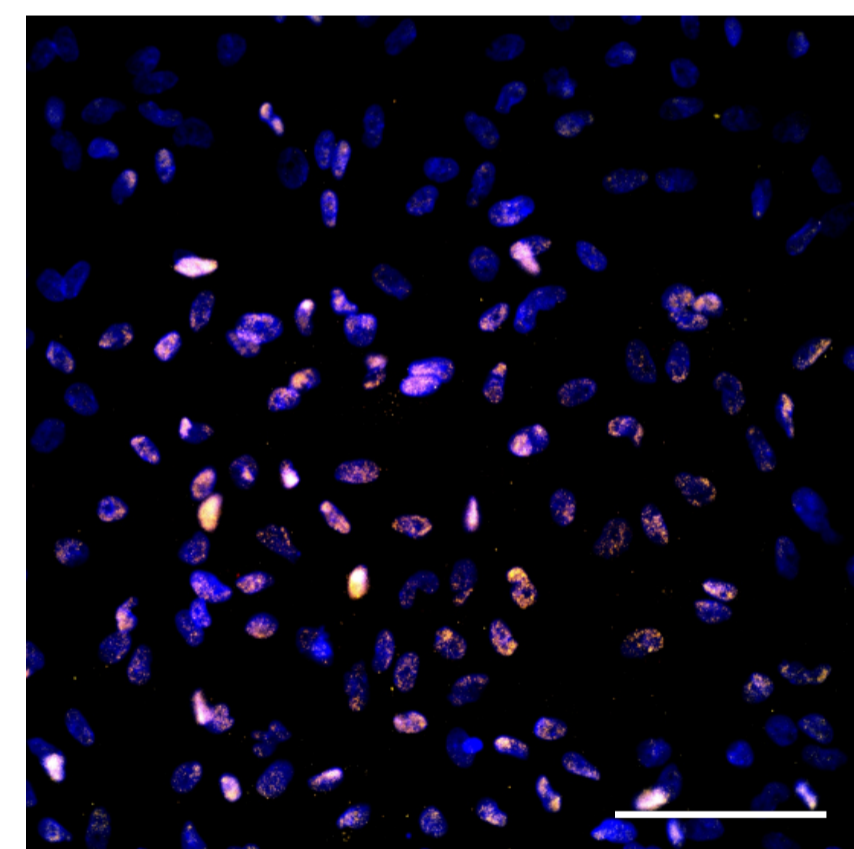

24 h

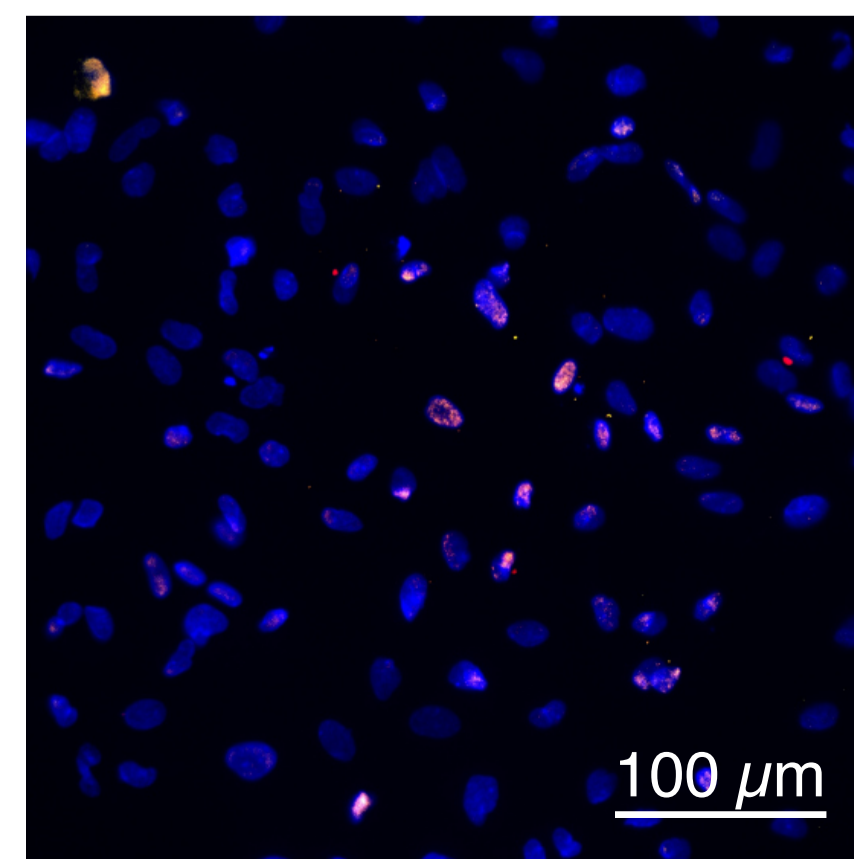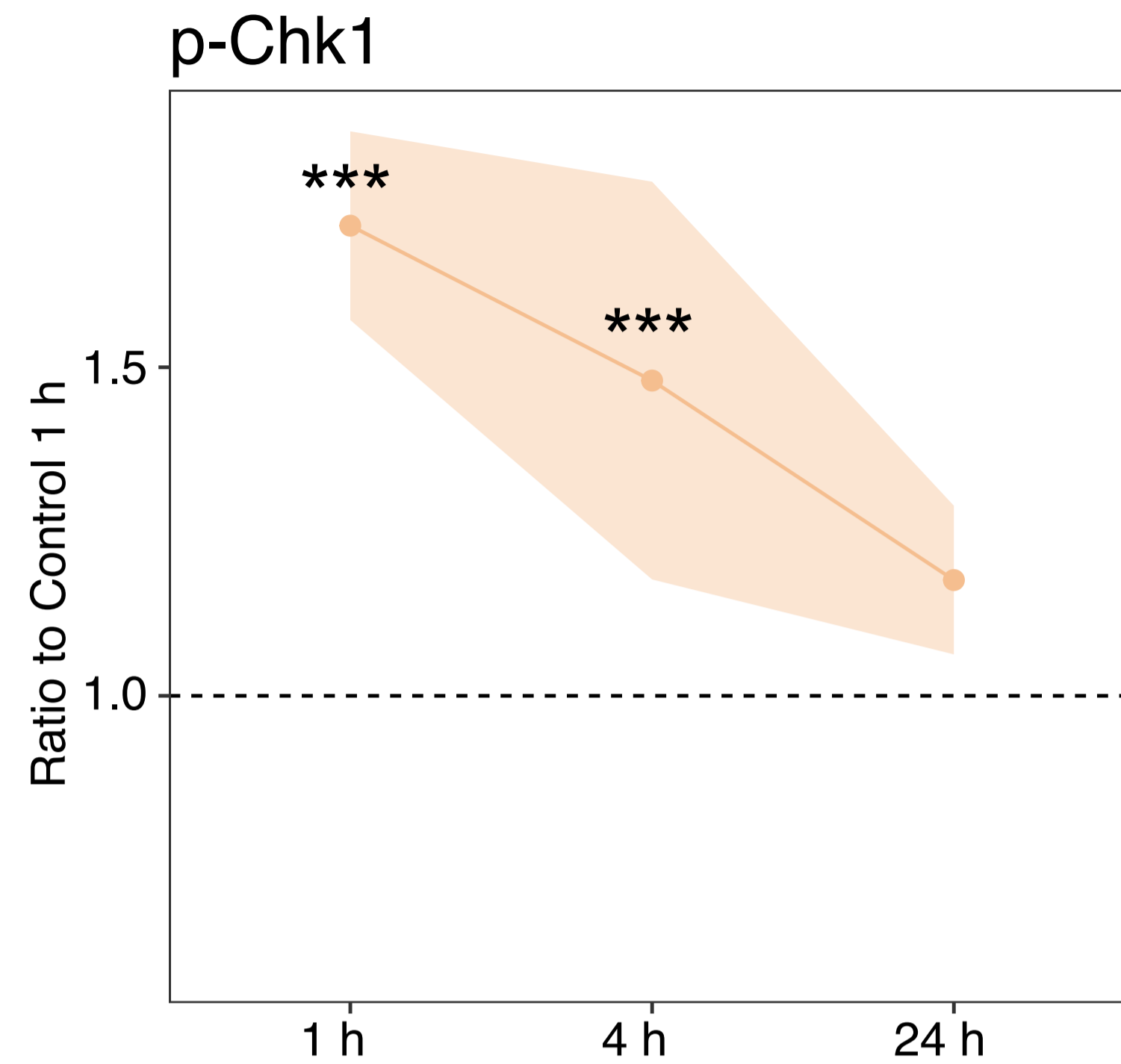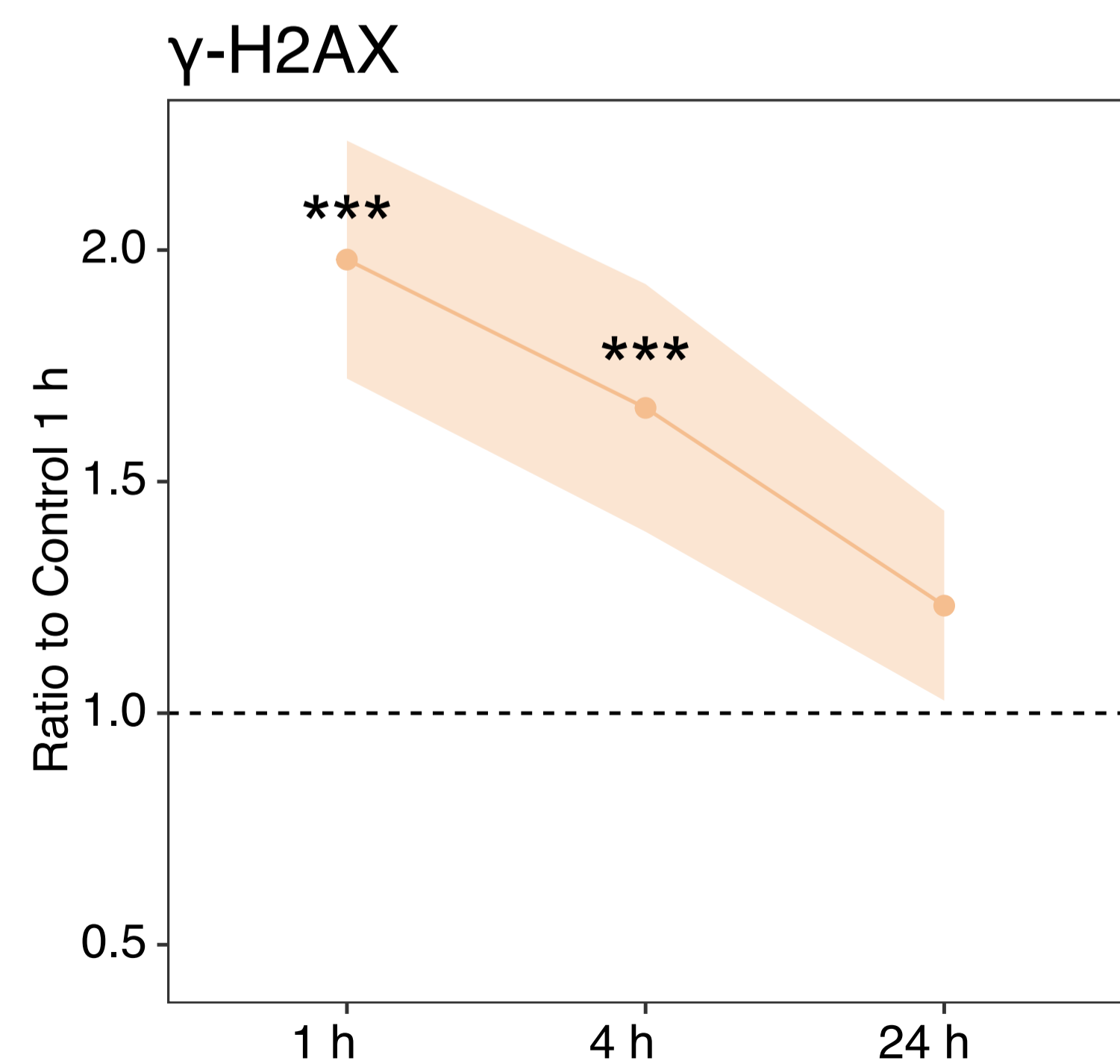

B

mPOCs

Control 1 h

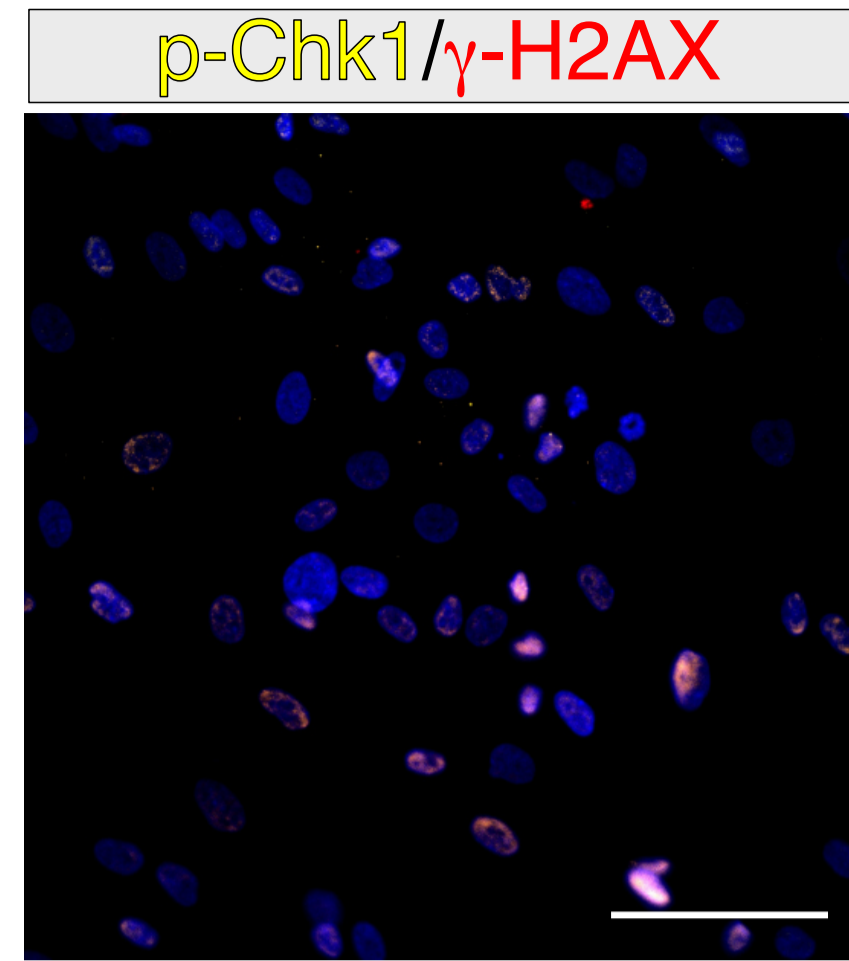

1 h

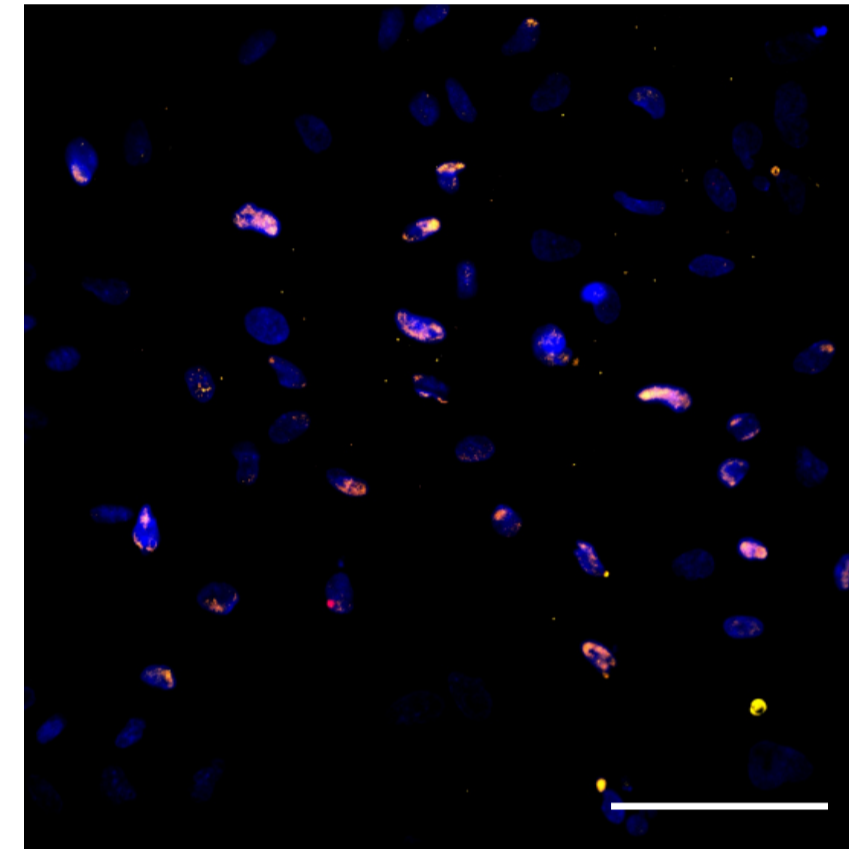

4 h

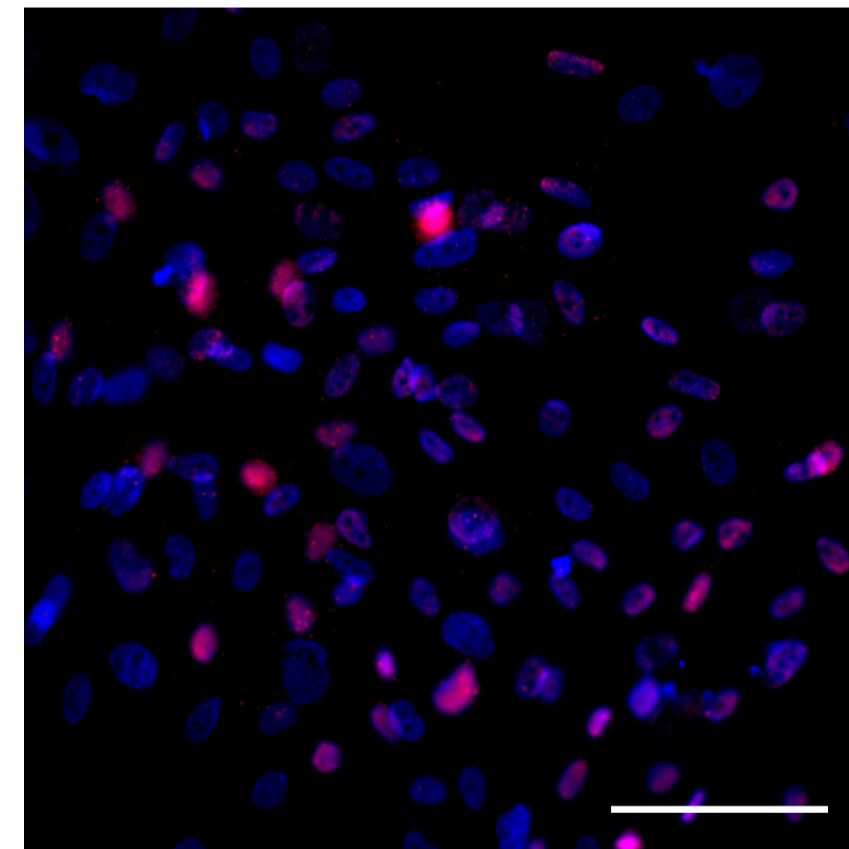

24 h

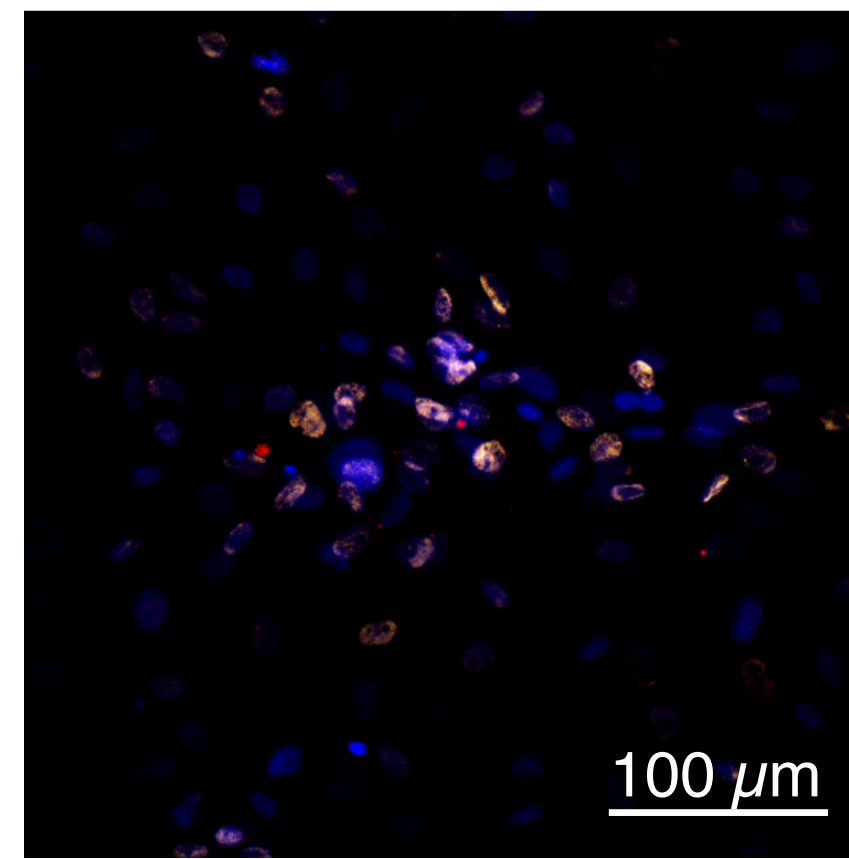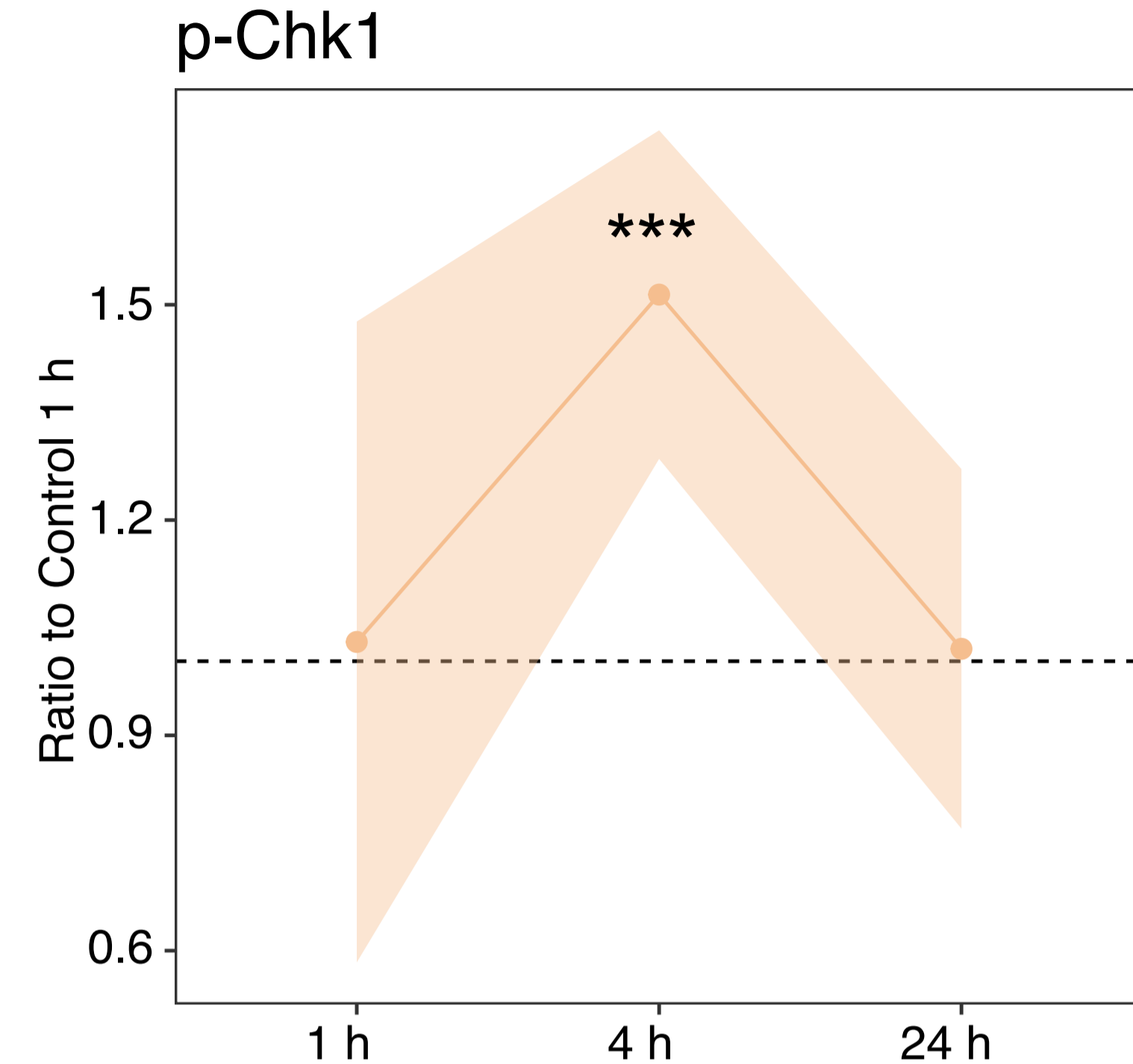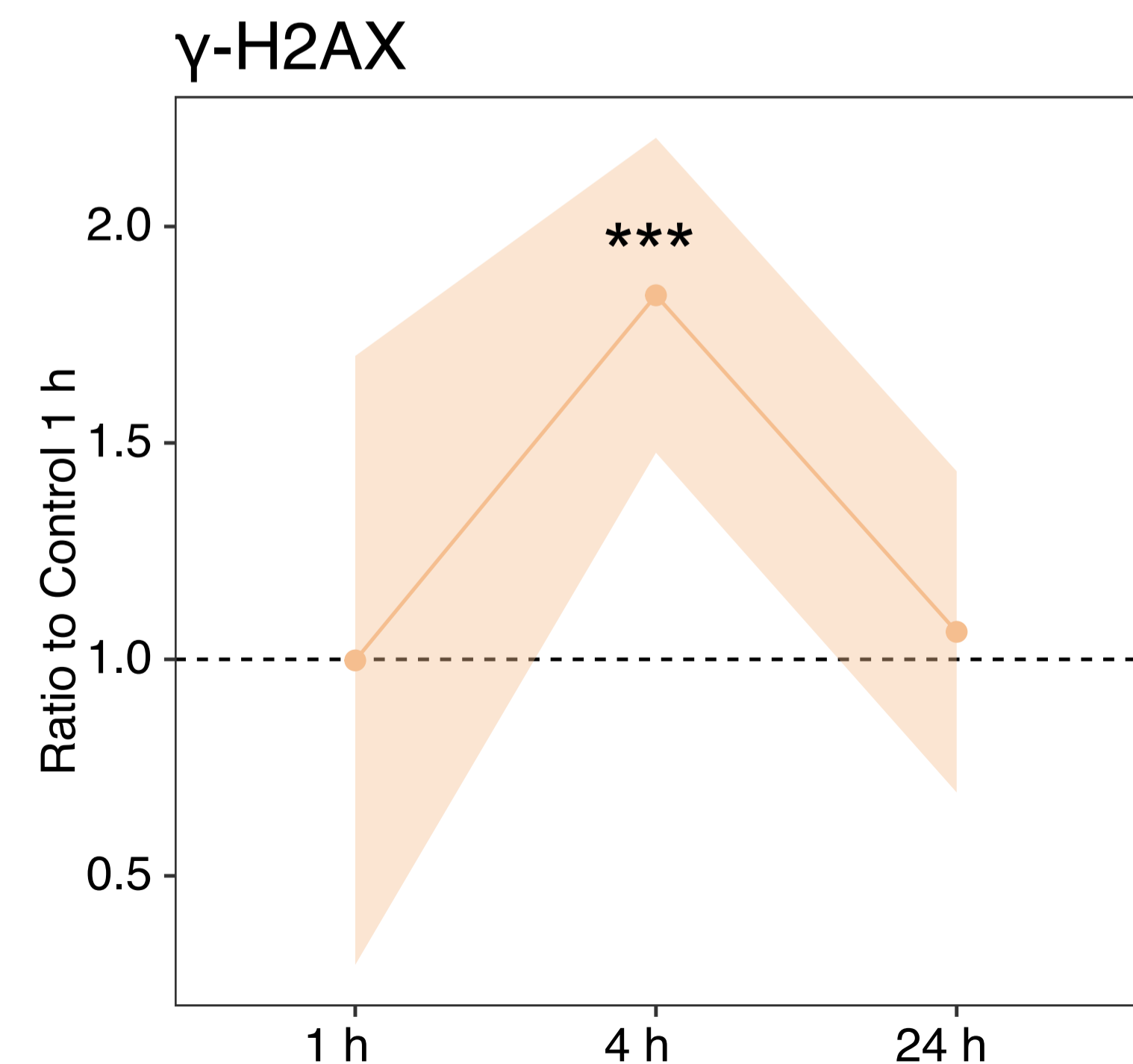

Supplement: Supplementary file 11 — Supplementary Material 11. [file 13048_2025_1932_MOESM11_ESM.pdf]

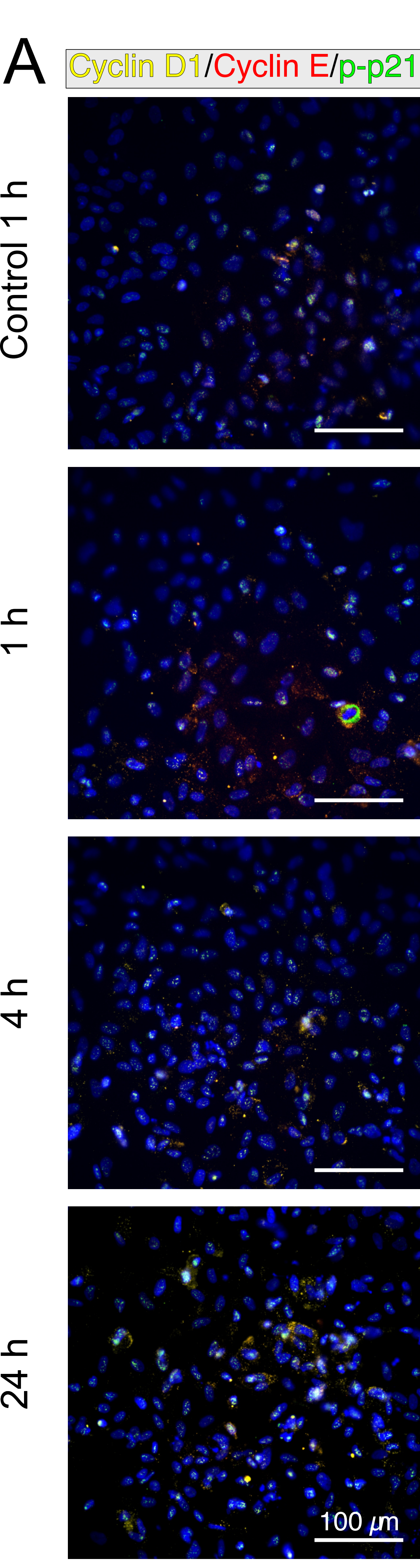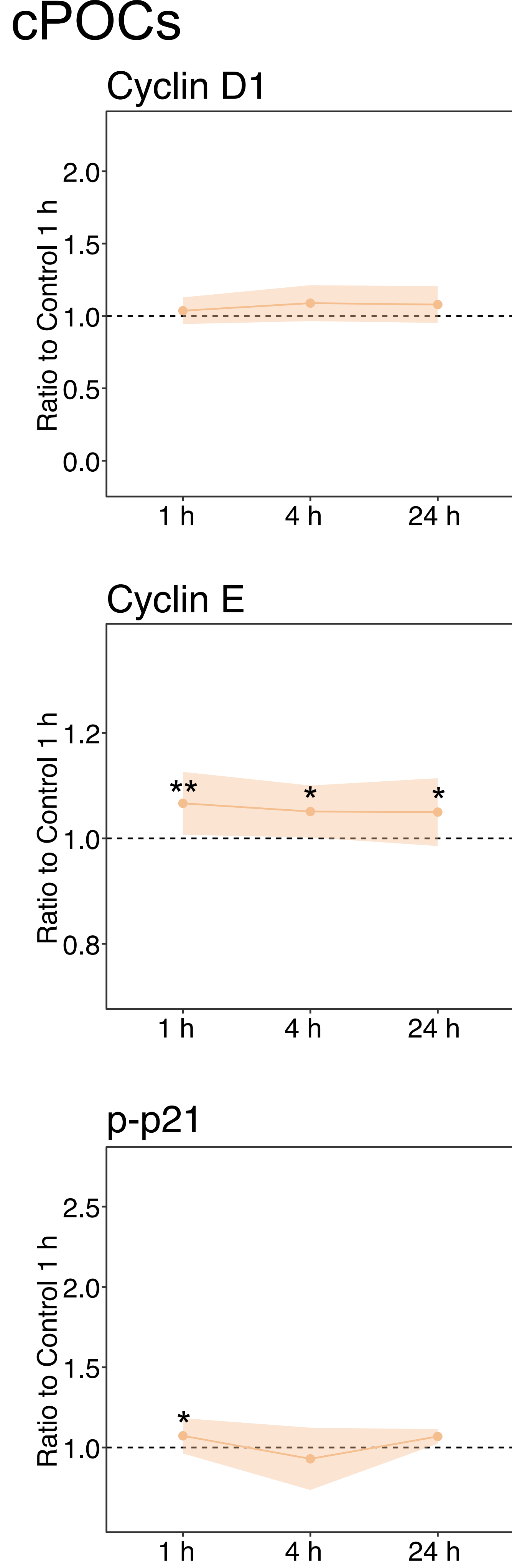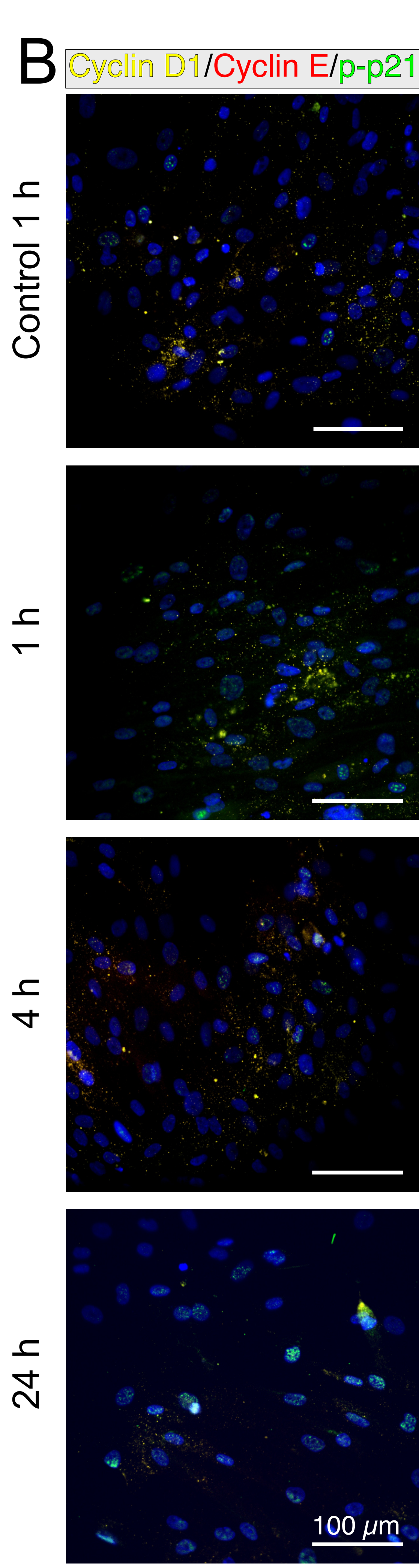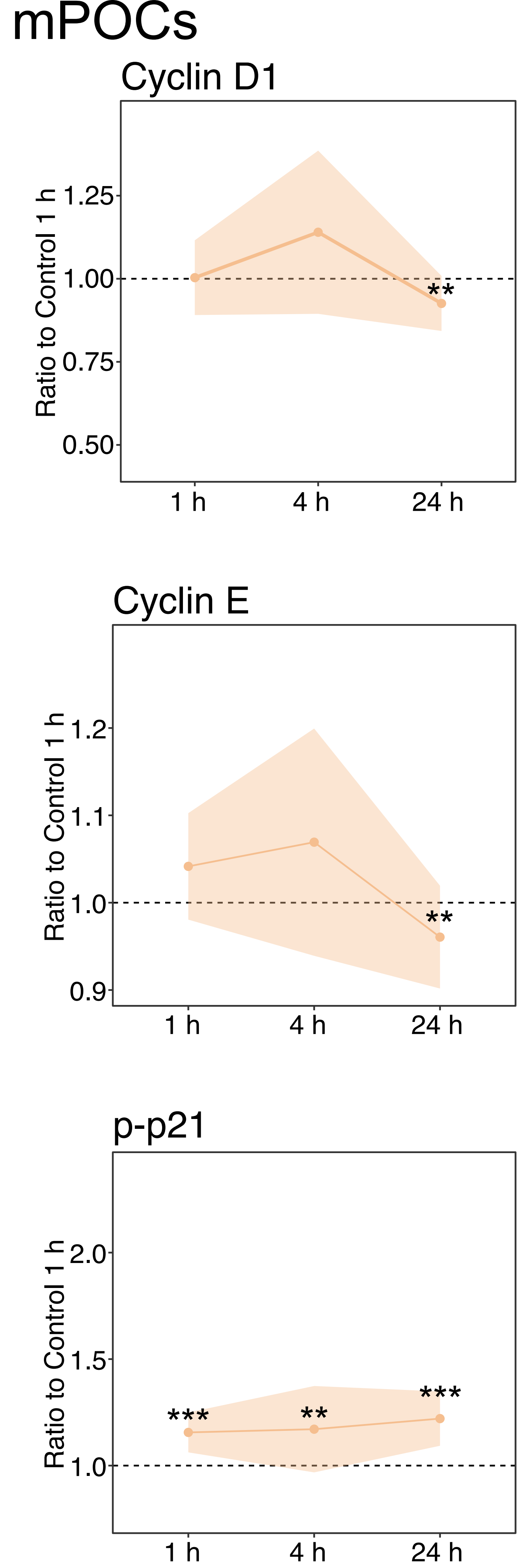

Supplement: Supplementary file 12 — Supplementary Material 12. [file 13048_2025_1932_MOESM12_ESM.pdf]

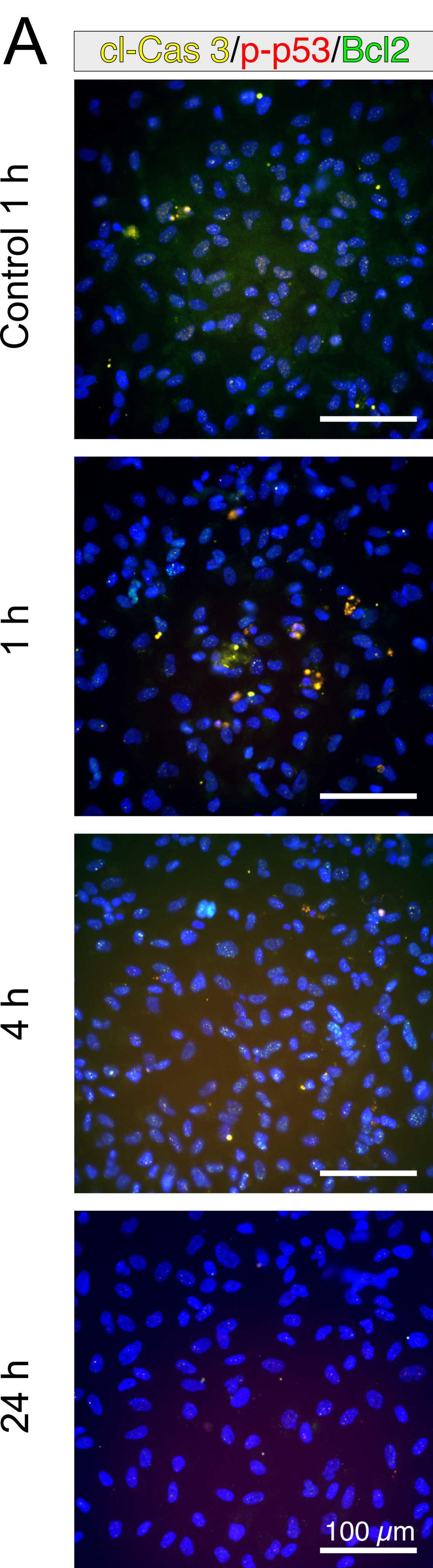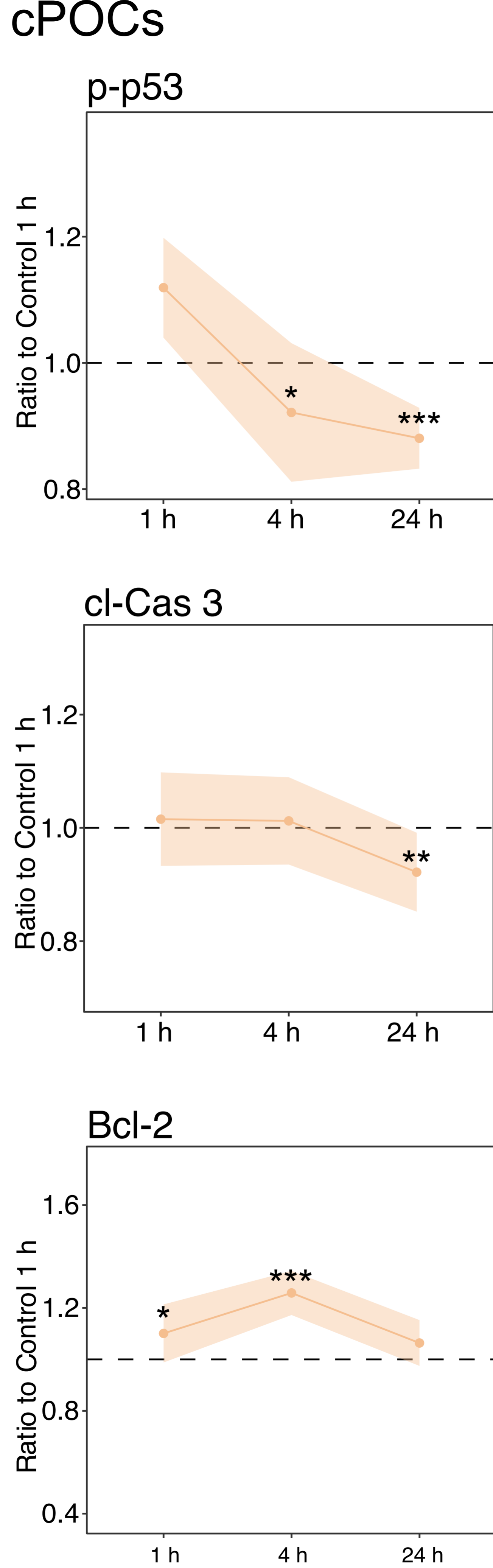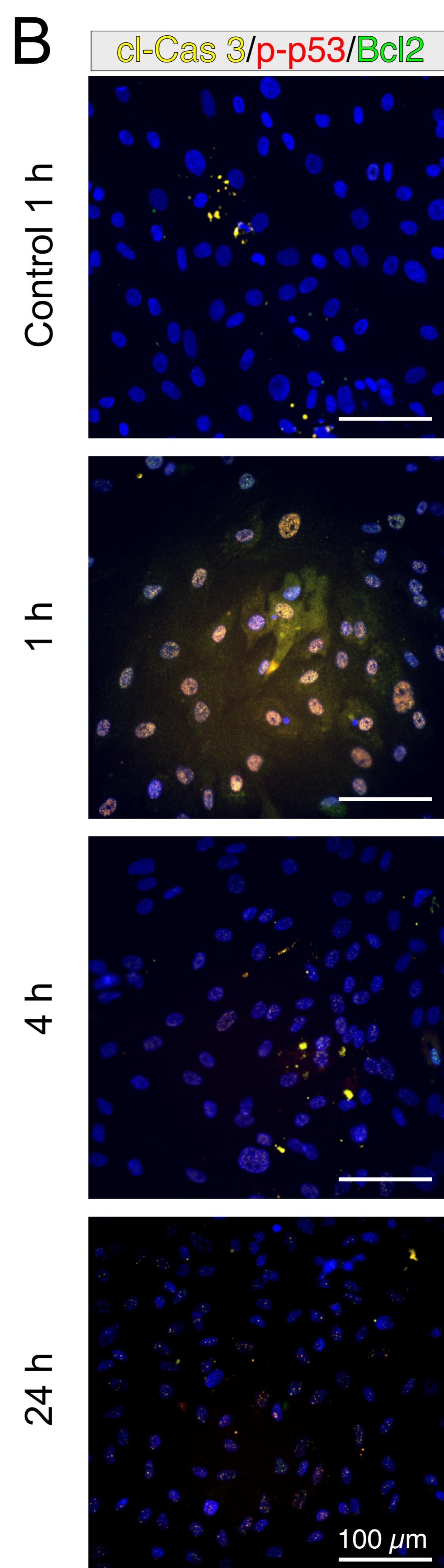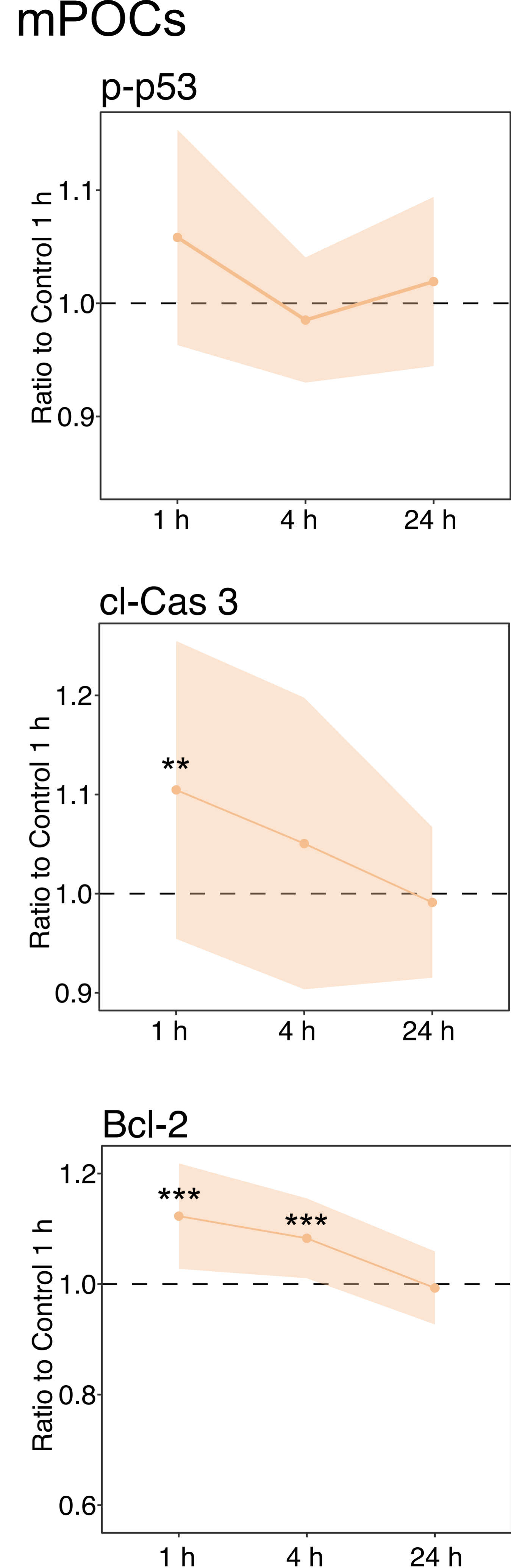

Supplement: Supplementary file 13 — Supplementary Material 13. [file 13048_2025_1932_MOESM13_ESM.pdf]

Control

cPOCs

mPOCs

BF

H&E

BF

H&E

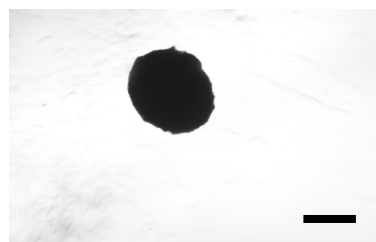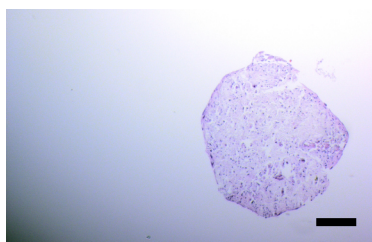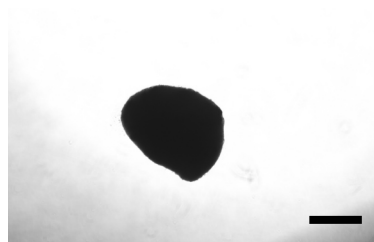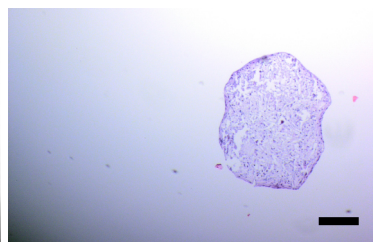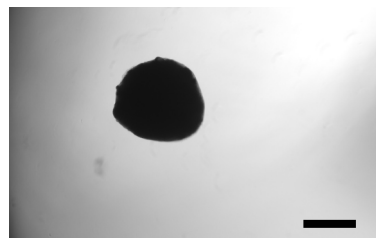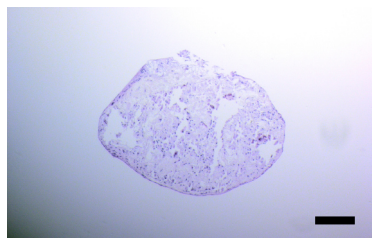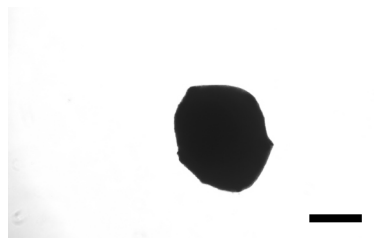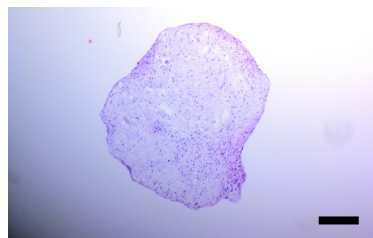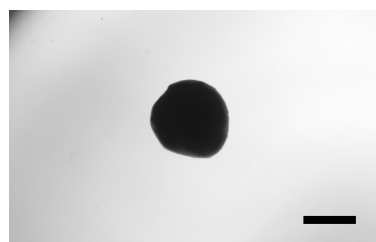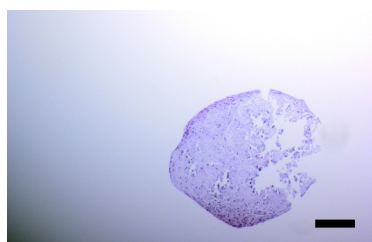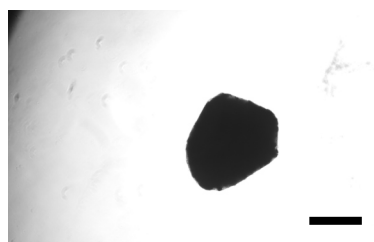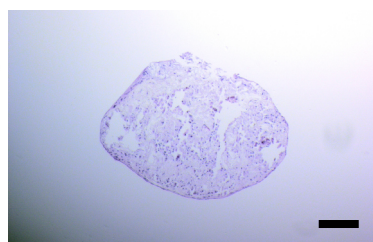

Irradiated

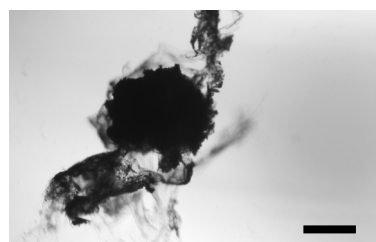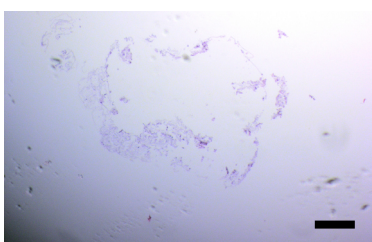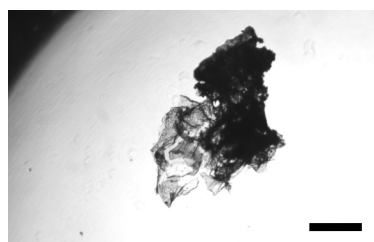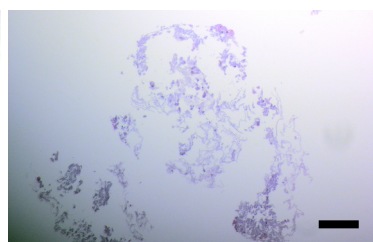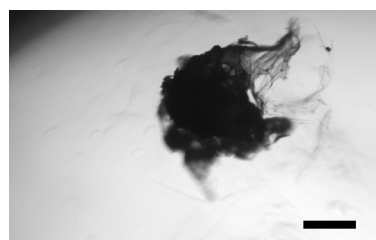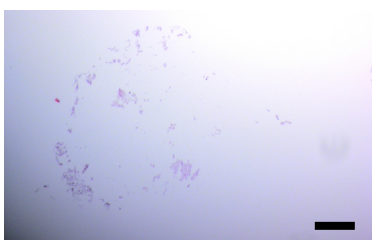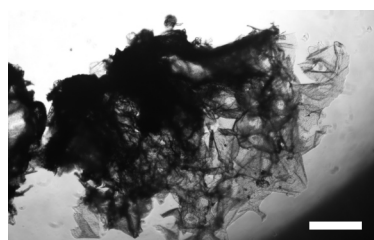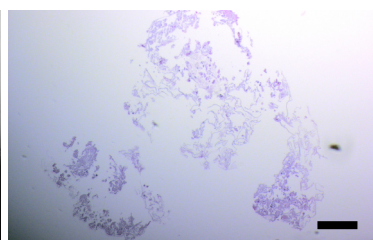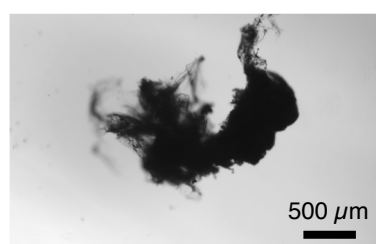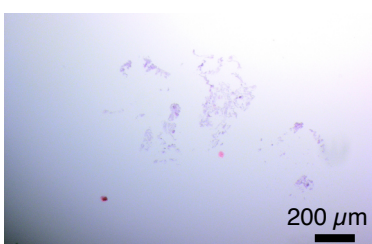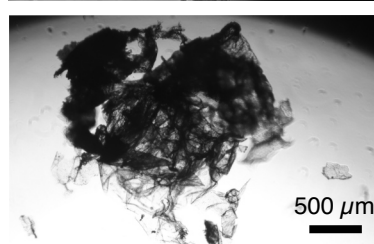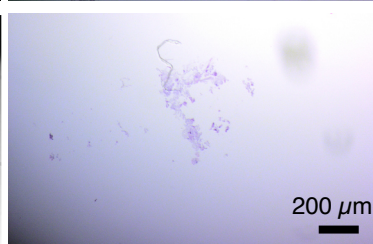

Supplement: Supplementary file 14 — Supplementary Material 14. [file 13048_2025_1932_MOESM14_ESM.pdf]

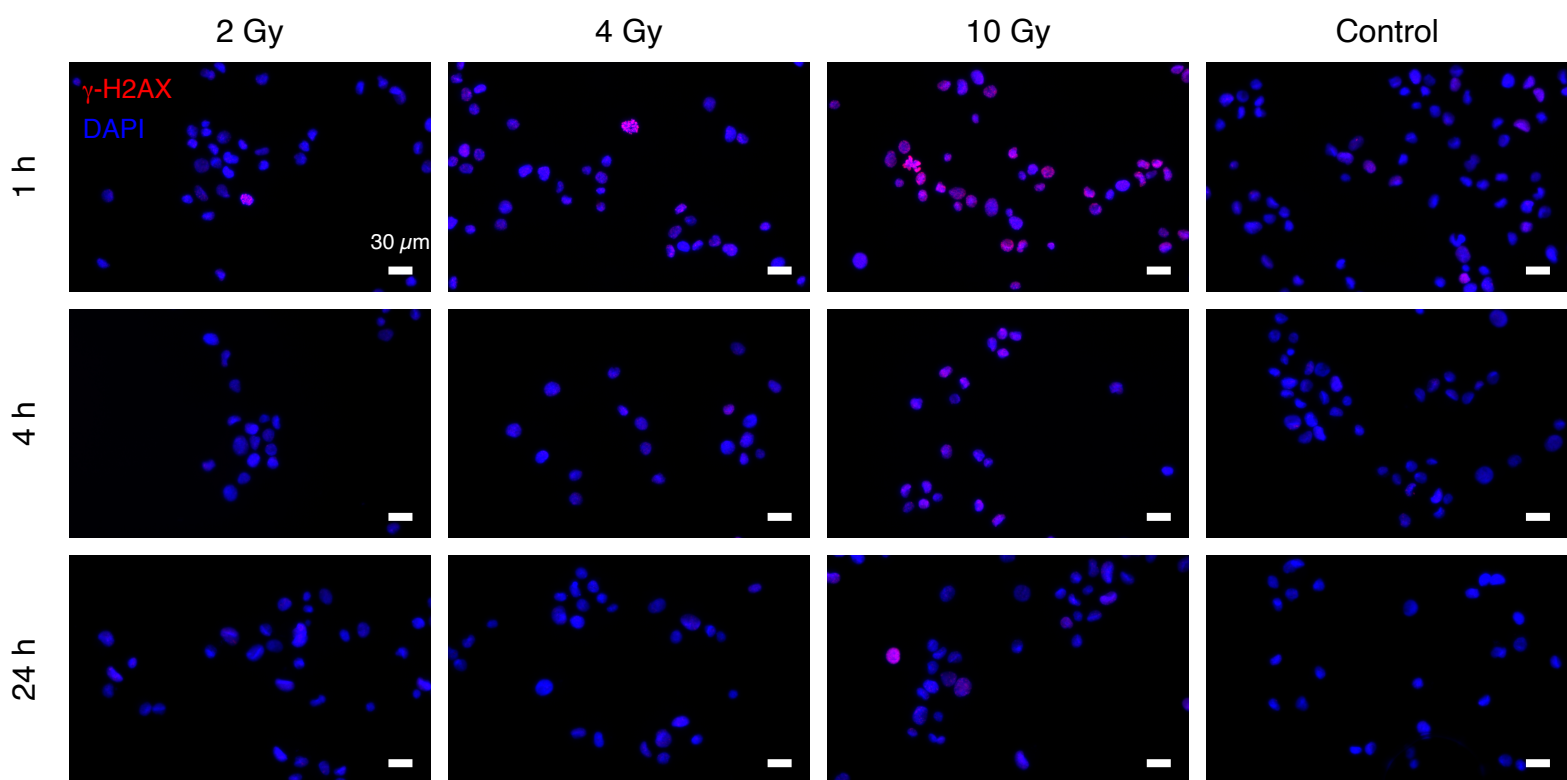

Supplement: Supplementary file 15 — Supplementary Material 15. [file 13048_2025_1932_MOESM15_ESM.pdf]
