# Supplementary material for: Acute high-dose irradiation disrupts cell adhesion and Silk-Ovarioid formation in human primary ovarian cells
Source: J Ovarian Res. 2026 Jan 2;19:79. doi: 10.1186/s13048-025-01932-8 (PMC12930946; doi:10.1186/s13048-025-01932-8)
Supplement: Supplementary file 10 — Supplementary Material 10. [file 13048_2025_1932_MOESM10_ESM.pdf]

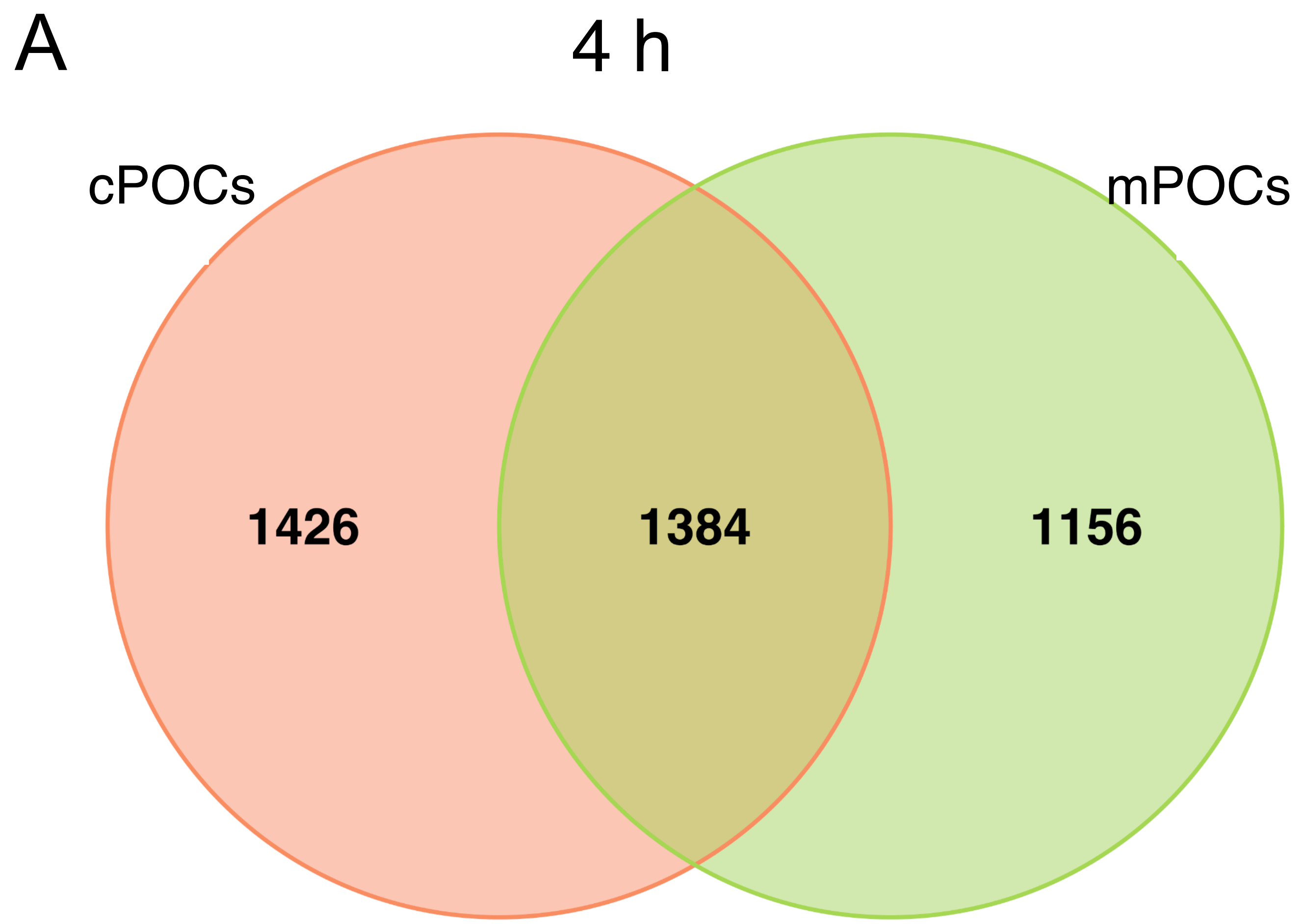

cPOCs specific GO	Overlapping GO	mPOCs specific GO
Ubiquitine ligase complex<br>Autophagosome organization	Nuclear speck<br>DNA-binding transcription repressor<br>Ubiquitin-protein transferase<br>Histone modifying activity<br>Aminoacyl-transferase activity	Extracellular matrix<br>Collagen<br>Extracellular matrix organization<br>Collagen fibril organization<br>Basement membrane

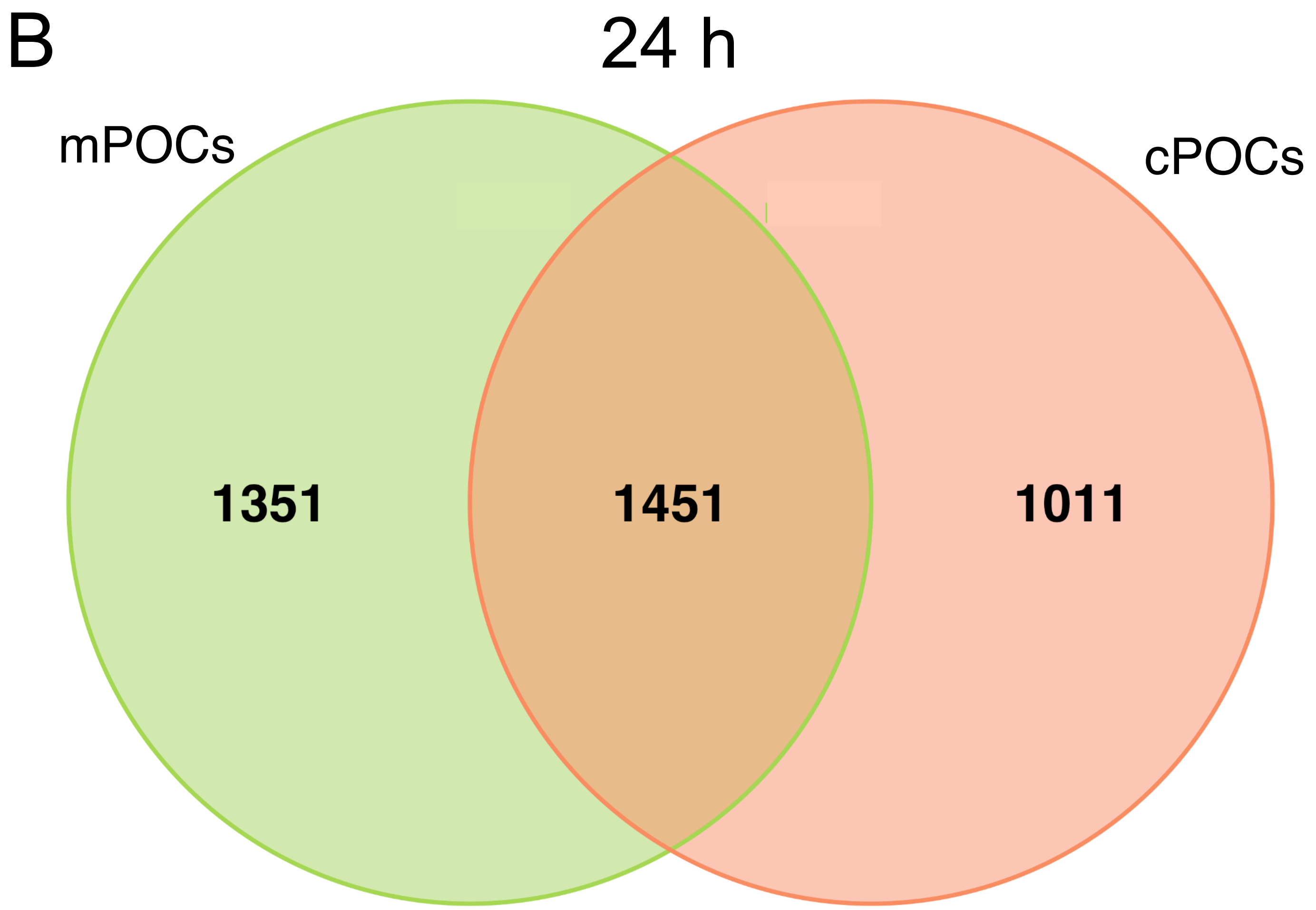

mPOCs specific GO	Overlapping GO	cPOCs specific GO
Angiogenesis<br>Epithelium migration<br>Cell-cell junction<br>Focal adhesion<br>Cell-substrate adhesion	Mitotic cell cycle phase transition<br>Cell cycle checkpoint signaling<br>DNA replication<br>Sister chromatid segregation<br>Organelle fission	DNA replication<br>Mitotic spindle<br>Regulation of fatty acid transport<br>Positive regulation of protein Ser/Thr kinase activity
